# Inhibition of DRP-1 mitochondrial mitophagy and fission by novel α-aminophosphonates bearing pyridine: synthesis, biological evaluations, and computer-aided design

**DOI:** 10.1186/s13065-024-01268-2

**Published:** 2024-09-18

**Authors:** Hend A. Hekal, Maha M. Salem, Hayam A. Abd El Salam

**Affiliations:** 1 Chemistry Department, Faculty of Science, Tanta University, Tanta, 31527 Egypt; 2 Biochemistry Division, Chemistry Department, Faculty of Science, Tanta University, Tanta, 31527 Egypt; 3 Green Chemistry Department, National Research Centre, Dokki, Giza, 12622 Egypt

**Keywords:** Mitochondrial fission, Dynamin-related protein 1, Antimicrobial, α-Aminophosphonates, Pyridine, Kabachnic-fields reaction

## Abstract

Heterocyclic compounds play a crucial role in the drug discovery process and development due to their significant presence and importance. Here, we report a comprehensive analysis of α-aminophosphonates containing pyridine (**3a**–**g**), prepared according to a clear-cut, uncomplicated procedure. The phosphonates are thoroughly characterized using various methods, such as elemental analysis, mass spectrometry, proton and carbon NMR, and FT-IR. The molecular docking interactions between the phosphonate and DRP-1 target protein observed that compound **3d** had the top-ranked binding energy towards DRP-1 with a value equal to − 9.54 kcal/mol and this theoretically proves its inhibitory efficacy against DRP-1 arbitrated mitochondrial fission. Besides, the anticancer characteristics of compound **3d** showed the best IC_50_ against HepG-2, MCF-7, and Caco-2 which confirmed our results towards suppressing DRP-1 protein (in-silico), and it elucidated no cytotoxic effects against human normal cell line (WI-38). Further, its pharmacokinetics were observed theoretically using ADMET. Moreover,compound **3d** investigated the most potent antimicrobial ability against two pathological fungal strains, *A. flavus* and *C. albicans*, and four bacterial strains, *E. coli*, *B. subtillis*, *S. aureus*, and *P. aregeunosa*. Additionally, compound **3d** clarified a powerful antioxidant scavenging activity against DPPH and ABTS free radicals (in-vitro). Furthermore, Density functional theory (DFT) was used to study the molecular structures of the synthesized compounds **3a**–**g**, utilizing 6–311++G(d,p) as the basis set and to learn more about the molecules’ reactive sites, the energies of the molecular electrostatic potential (MEP), the lowest unoccupied molecular orbital (LUMO), and the highest occupied molecular orbital (HOMO) were observed. Theoretically, FT-IR and Nuclear magnetic resonance (NMR) measurements are calculated for every compound under investigation to show how theory and experiment relate. It was found that there was an excellent agreement between the theoretical and experimental data. Conclusively, all novel synthesized phosphonates could be used as pharmaceutical agents against pathogenic microbial strains and as anticancer candidates by inhibiting DRP-1-mediated mitochondrial mitophagy.

**Supplementary Information:**

The online version contains supplementary material available at 10.1186/s13065-024-01268-2.

## Introduction

The development of heterocyclic chemistry and its effective application in medicinal chemistry transformed the process of finding new drugs. Organophosphorus compounds have attracted intensely expanding interest and exciting applications in agricultural and industrial fields during the past few decades as an important class in organic synthesis, medicinal chemistry, and biological activities [1–3]. The α-aminophosphonates, also known as bio isosteres of amino acids, are extensively explored as fascinating organophosphorus derivatives [4]. They possess promising biological and pharmacological potentials as antioxidants [5, 6], antibiotics [7, 8], antiviral [9, 10], antitumoral [9, 11], and anti-inflammatory [6].

**Fig. 1:**
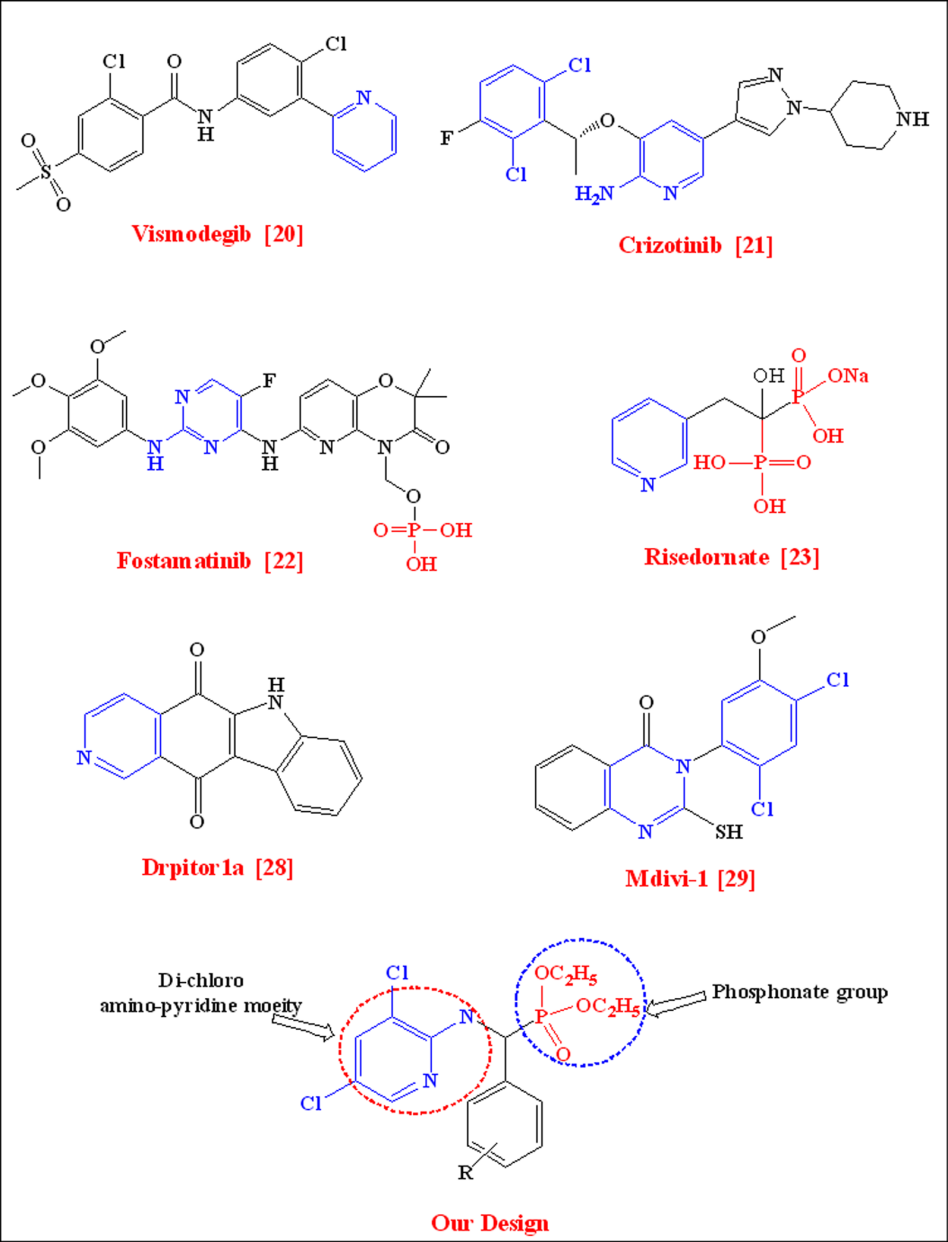
Biomedical drugs containing pyridine and phosphonic acidscaffolds

Furthermore, the pyridine moiety is crucial in finding novel medications. It is present in many medically relevant chemicals utilized in the pharmaceutical industry, such as anesthetics, prodrugs to cure neuronal damage, and anti-cancer, and anti-inflammatory medications for some brain illnesses [12]. Notably, the pyridine derivatives with amino, chloro, and trifluoromethyl groups have been frequently reported for their anticancer activity [13]. As a result, numerous heterocyclic nuclease 2-amino pyridine derivatives have demonstrated strong pharmacological effects [14–18]. As per the above summary, certain pharmaceutical companies employed pyridine and phosphonates as efficacious medications, including, **Vismodegib** was approved by the US Food and Drug Administration (FDA) for the treatment of basal cell carcinoma (BCC) [19], **Crizotinib**, a small-molecule kinase inhibitor, which the (we) FDA approved for the treatment of patients with locally advanced or metastatic non-small-cell lung cancer [20], **Fostamatinib**, is a spleen tyrosine kinase inhibitor used to treat chronic immune thrombocytopenia [21], and **Risedronate**, which is an effective and well-tolerated therapy for the treatment of postmenopausal osteoporosis [22], Fig. 1.

It is well-established that mitochondrial dynamics plays a significant part in age-related disorders, such as cancer. Nevertheless, studies on mitochondrial dynamics and cancer are still in their early stages of discovery. Organelles called mitochondria are involved in various essential cellular processes, including the production of adenosine triphosphate (ATP), the processes by which cells divide and differentiate, the anabolic and catabolic functions of cells, and the breakdown of cells. In response to physiologic or stress-related stimuli, mitochondria modify their structure and function, which are closely related [23]. The important chemicals and mechanisms that influence or cause some of these structural and functional alterations have been the subject of extensive research in recent years. Mitochondrial fission and fusion occur in both normal cells and dysregulated cells, like cancer cells. These events may be the most significant of these structural alterations. Enough mitochondria are produced via mitochondrial fission to facilitate the division and growth of cells. In addition to producing new organelles, mitochondrial fission serves as a quality control mechanism by removing defective mitochondria via a process known as mitophagy. On the other hand, when mitochondria must rely on oxidative phosphorylation or respond to stress stimuli, they must fuse to produce the maximum amount of ATP. In these situations, they manifest as elongated, healthy organelles that complement the malfunctioning mitochondria [24].

The essential part of the mitochondrial fission machinery is dynamin-related protein 1 (Drp-1), a member of the dynamin family of guanosine triphosphatases (GTPases) [25]. Dynamin-related protein 1 has been connected to the emergence of several cancerous tumors. Dynamin-related protein 1 was involved in alterations to cellular metabolism, metastases, sustaining cell cycle, and proliferation. It was also associated with the oncolytic phenotype. Also, Drp-1 affects metabolic regulation in addition to a variety of other cell functions, including apoptosis, mitophagy and mitochondrial biogenesis, cell division, and transformation [26]. **Drpitor1a** [27], which is a pyridine derivative, is a potent DRP-1 inhibitor that prevents mitochondrial fission, Fig. 1. Another important DRP-1 inhibitor is Mitochondrial division inhibitor 1 (mdivi-1) [28], which contains di chloro substituents that decrease cancer cell proliferation by inducing mitochondrial fusion and altering oxygen consumption, Fig. 1. Mdivi-1 is the currently accepted gold standard FDA inhibitor of Drp-1, discovered by Cassidy-Stone et al. [29]. However, there was many limitations have been raised regarding the mdivi-1 [30, 31]. Therefore, there is an urgent need to find more accessible, nontoxic, potent, and specific Drp-1 GTPase inhibitors to be targeted for cancer treatment. We consider that the presence of pyridine group and dichloro groups in the skeleton of α-aminophosphonates may play a vital role in inhibition of dynamin-related protein 1 (DRP-1).

Computational chemistry advancements and knowledge of biological and biochemical structures and reactions have come together recently to create a comprehensive companion [32]. It has significantly advanced our knowledge of molecular characteristics, structure, and reaction selectivity [32]. In addition, it provides us with essential details about the compounds we are studying, such as total energy, dipole moment, electronic energy, binding energy, bond lengths, HOMO, and LUMO. This information’s high worth rises when it aligns with experimental results. It also aids in our comprehension of how the molecule will probably behave during reactions [33]. Density functional theory (DFT) is the most promising approach for applying computer-based calculations to study the electrochemical characteristics of various substances [6, 11, 33, 34].

Considering these, we attempted to create a novel class of α-aminophosphonates with pyridine nucleus and investigated their antimicrobial, antioxidant, and anticancer impact via elucidatingtheir inhibitory role on DRP-1 mediated mitochondria fission (in-silico and in-vitro). Additionally, using the DFT technique, the effect of modifications to the electrical and molecular structures on the biological activity of freshly synthesized compounds was examined. Further, their bioavailability and drug-likeness were examined using ADMET.

## Experimental

### Materials and instrumentation

The chemical and instrument data are all contained in the supplementary file (Section S1).

### Synthesis of α-aminophosphonate compounds 3a–g

Triethyl phosphite was added together with anhydrous lithium perchlorate LiClO_4_ (10 mol%) to a stirred mixture of 3,5-dichloropyridin-2-amine **1** (0.01 mol) and suitable aldehyde derivatives **2a**–**g** (0.0012 mol) in dry dichloromethane CH_2_Cl_2_. TLC results showed that the reaction was finished after 72–84 h of stirring at room temperature. After that, CH_2_Cl_2_ was evaporated, and cold methanol was used to precipitate the α-aminophosphonates. The precipitate was removed by filtering, and then recrystallize from ethanol producing fresh α-aminophosphonates **3a-g** in a very good yield.

#### Diethyl(((3,5-dichloropyridin-2-yl)amino)(4-hydroxy-3-methoxyphenyl)methyl)phosphonate, 3a

Yield (1.11 gm, 85%); 72 h; yellow solid; M.p.: 66–68 °C; TLC: Ethyl acetate: Petroleum ether 1:3); Rf: 0.28; IR: ν/cm^−1^: 3248 (NH), 3029 (CH–Arom.), 2925 (CH-Aliph), 1254 (P=O), 1086 (P–O–C), 761 (P–CH). ^1^H NMR (500 MHz, DMSO-*d6*): *δ* = 3.66 (s, 3H, OCH_3_), 1.18 (t, 6H, *J* = 6.0 Hz, 2xCH_3_), 3.81–4.02 (m, 4H, 2xCH_2_), 6.46 (s, 1H, CH–P),6.66(1H, OH, exchangeable with D_2_O), 7.89 (s, 1H, H_pyridine_), 9.74 (s, 1H, H_pyridine_), 7.69 (s, 1H, H_arom_), 6.92 (d, 1H, *J* = 8.5 Hz, H_arom_), 7.35 (d, 1H, *J* = 9.0 Hz, H_arom_), 10.210 (s, 1H, NH, exchangeable with D_2_O) ppm. ^13^C NMR (125 MHz, DMSO-*d6*): *δ* 14.23 (2CH_3_), 62.24 (2CH_2_), 68.74 (CH–P), 56.35 (O–CH_3_), 153.49 (C=N pyridine), 154.95 (C–NH), 111.11–148.78 (Ar–C) ppm. MS (EI) *m*/*z*: 436 (M+, 3%, C_17_H_21_Cl_2_N_2_O_5_P), 298 (M−3, 50%, C_13_H_12_Cl_2_N_2_), 161(100%, C_5_H_4_Cl_2_N_2_), 136 (M−2, 28%, C_4_H_11_O_3_P). Anal. Calcd. For C_17_H_21_Cl_2_N_2_O_5_P (434.06): C, 46.91; H, 4.86; N, 6.44; P, 7.12. Found;C, 46.88; H, 4.81; N, 6.38; P, 7.11.

#### Diethyl (((3,5-dichloropyridin-2-yl) amino)(4-hydroxy phenyl) methyl)phosphonate, 3b

Yield (0.97 gm, 80%); 80 h; buff solid; M.p.: 56–58 °C; TLC: Ethyl acetate: Petrolum ether 1:3); Rf: 0.25; IR: ν/cm^−1^: 3248 (NH), 3029 (CH–Arom.), 2925 (CH–Aliph), 1254 (P=O), 1086 (P–O–C), 761 (P–CH). ^1^H NMR (500 MHz, DMSO-*d6*): *δ* = 1.20 (t, 6H, *J* = 6.5 Hz, 2xCH_3_), 3.97–4.03 (m, 4H, 2xCH_2_), 6.09 (s, 1H, CH–P), 6.47(1H, OH, exchangeable with D_2_O), 7.89 (s, 1H, H_pyridine_), 9.76 (s, 1H, H_pyridine_), 6.89 (d, 2H, *J* = 7.5 Hz, H_arom_), 7.72 (d, 2H, *J* = 9.5 Hz, H_arom_), 10.56 (s, 1H, NH, exchangeable with D_2_O) ppm.^13^C NMR (125 MHz, DMSO-*d6*): *δ* 27.72 (2CH_3_), 58.38 (2CH_2_), 69.91 (CH–P), 155.04 (C=N pyridine), 163.81 (C–NH), 114.29–142.14 (Ar–C) ppm. MS (EI) *m*/*z*: 403 (M−, 7%, C_16_H_19_Cl_2_N_2_O_4_P), 270 (M+1, 8%, C_12_H_10_Cl_2_N_2_O), 161(100%, C_5_H_4_Cl_2_N_2_), 136 (M−2, 28%, C_4_H_11_O_3_P); Anal. Calcd. For C_16_H_19_Cl_2_N_2_O_4_P (404.05):C, 47.43; H, 4.73; N, 6.91; P, 7.64.Found;C, 47.40; H, 4.69; N, 6.85; P, 7.62.

#### Diethyl (((3,5-dichloropyridin-2-yl)amino)(2-methoxyphenyl)methyl)phosphonate, 3c

Yield (1.04 gm, 83%); 72 h; pale yellow solid; M.p.: 73–75 °C; TLC: Ethyl acetate: Petrolum ether 1:3); Rf: 0.23;IR: ν/cm^−1^: 3248 (NH), 3029 (CH–Arom.), 2925 (CH–Aliph), 1254 (P=O), 1086 (P–O–C), 761 (P–CH). ^1^H NMR (500 MHz, DMSO-*d6*): *δ* = 3.75 (s, 3H, OCH_3_), 0.97–1.18 (m, 6H, 2xCH_3_), 3.83–3.99 (m, 4H, 2xCH_2_), 5.93 (d, 1H, *J* = 6 Hz, CH–P),7.23 (s, 1H, H_pyridine_), 8.04 (s, 1H, H_pyridine_), 6.48–7.90 (m, 4H, H_arom_),10.34 (s, 1H, NH, exchangeable with D_2_O) ppm.^13^C NMR (125 MHz, DMSO-*d6*): *δ* 17.11 (2CH_3_), 62.61 (2CH_2_), 63.89 (CH–P), 56.02 (O–CH_3_), 144.64 (C=N pyridine), 155.06 (C–NH), 111.12–136.66 (Ar–C) ppm. MS (EI) *m*/*z*: MS (EI) *m*/*z*: 419 (M+1, 9%, C_17_H_21_Cl_2_N_2_O_4_P), 280 (M−2, 100%, C_13_H_12_Cl_2_N_2_O), 177 (100%, C_6_H_6_Cl_2_N_2_), 136 (M−2, 87%, C_4_H_11_O_3_P); Anal. Calcd. For C_17_H_21_Cl_2_N_2_O_4_P (418.06): C, 48.70; H, 5.05; N, 6.68; P, 7.39.Found;C, 48.68; H, 5.03; N, 6.62; P, 7.35.

#### Diethyl (((3,5-dichloropyridin-2-yl)amino)(2-hydroxynaphthalen-1-yl)methyl)phosphonate, 3d

Yield (1.16 gm, 85%); 76 h; dark yellow solid; M.p.: 145–147 °C; TLC: Ethyl acetate: Petrolum ether 1:1); Rf: 0.30; IR: ν/cm^−1^: 3248 (NH), 3029 (CH–Arom.), 2925 (CH–Aliph), 1254 (P=O), 1086 (P–O–C), 761 (P–CH). ^1^H NMR (500 MHz, DMSO-*d6*): *δ* = 1.00, 1.200 (2 × t, 6H, *J* = 8 Hz, 2xCH_3_), 3.84–4.02 (m, 4H, 2xCH_2_), 5.72 (d, 1H, *J* = 9 Hz, CH–P),7.73 (s, 1H, H_pyridine_), 8.91 (s, 1H, H_pyridine_), 7.19 (d, 1H, *J* = 9.5 Hz, H_arom_), 7.39 (d, 1H, *J* = 8.2 Hz, H_arom_), 7.56–8.15 (5H, s, 1H, OH, m, 4H, H_arom_), 10.78 (br, 1H, NH, exchangeable with D_2_O) ppm.^13^C NMR (125 MHz, DMSO-*d6*): *δ* = 16.20 (2CH_3_), 60.22 (2CH_2_), 70.03 (CH–P), 144.17 (C=N pyridine), 164.23 (C–NH), 112.78–139.14 (Ar–C) ppm. MS (EI) *m*/*z*: 455 (M+1, 5%, C_20_H_21_Cl_2_N_2_O_4_P), 312 (M+3, 8%, C_10_H_15_Cl_2_N_2_O_3_P), 177 (3%, C_6_H_6_Cl_2_N_2_), 161(27%, C_5_H_4_Cl_2_N_2_), 146 (M+2, 38%, C_10_H_8_O), 136(M−2, 18%, C_4_H_11_O_3_P); Anal. Calcd. For C_20_H_21_Cl_2_N_2_O_4_P (454.06):C, 52.76; H, 4.65; N, 6.15; P, 6.80. Found;C, 52.73; H, 4.61; N, 6.12; P, 6.77.

#### Diethyl (((3,5-dichloropyridin-2-yl)amino)(4-(dimethylamino)phenyl)methyl)phosphonate, 3e

Yield (1.06 gm, 82%); 79 h; red solid; M.p.: 70–72 °C; TLC: Ethyl acetate: Petroleum ether 1:3); Rf: 0.24; IR: ν/cm^−1^: 3248 (NH), 3029 (CH–Arom.), 2925 (CH-Aliph), 1254 (P=O), 1086 (P–O–C), 761 (P–CH). ^1^H NMR (500 MHz, DMSO-*d6*): *δ* = 1.20 (t, 6H, *J* = 7 Hz, 2xCH_3_), 3.97–4.03 (m, 4H, 2xCH_2_), 6.47 (s, 1H, CH–P),7.89 (s, 1H, H_pyridine_), 9.63 (s, 1H, H_pyridine_), 6.72 (d, 2H, *J* = 8.6 Hz, H_arom_), 7.63 (d, 2H, *J* = 8.7 Hz, H_arom_), 2.99 (s, 6H, N(CH_3_)_2_) ppm. ^13^C NMR (125 MHz, DMSO-*d6*): *δ* = 16.72 (2CH_3_), 61.88 (2CH_2_), 72.06 (CH–P), 44.57 (N–(CH_3_)_2_), 144.62 (C=N pyridine), 154.73 (C–NH), 108.70–136.47 (Ar–C) ppm. MS (EI) *m*/*z*: 432 (M+1, 3%, C_18_H_24_Cl_2_N_3_O_3_P), 295 (M−1, 2%, C_14_H_15_Cl_2_N_3_), 177 (3%, C_6_H_6_Cl_2_N_2_), 161(M, 9%, C_5_H_4_Cl_2_N_2_), 148 (M+2, 100%, C_5_H_3_Cl_2_N), 136 (M−2, 18%, C_4_H_11_O_3_P); Anal. Calcd. For C_18_H_24_Cl_2_N_3_O_3_P (431.09): C, 50.01; H, 5.60; N, 9.72; P, 7.17. Found;C, 49.97; H, 5.56; N, 9.70; P, 7.12.

#### Diethyl (((3,5-dichloropyridin-2-yl)amino)(thiophen-3-yl)methyl)phosphonate, 3f

Yield (1.22 gm, 82%); 76 h; light brown solid; M.p.: 88–90 °C;TLC: Ethyl acetate: Petroleum ether 1:3); Rf: 0.25; IR: ν/cm^−1^: 3248 (NH), 3029 (CH–Arom.), 2925 (CH–Aliph), 1254 (P=O), 1086 (P–O–C), 761 (P–CH). ^1^H NMR (500 MHz, DMSO-*d6*): *δ* = 1.06–1.21 (m, 6H, 2xCH_3_), 3.91–3.99 (m, 4H, 2xCH_2_), 5.18 (d, 1H, *J* = 6.5 Hz, CH–P), 7.71 (s, 1H, H_pyridine_), 8.07 (s, 1H, H_pyridine_), 7.89 (s, 1H, H_thiophene_), 6.47–7.44 (m, 2H, H_arom_)ppm. ^13^C NMR (125 MHz, DMSO-*d6*): *δ* = 13.56 (2CH_3_), 57.45 (2CH_2_), 63.81 (CH–P), 144.70 (C=N pyridine), 154.09 (C–NH), 99.80–136.79 (Ar–C) ppm. MS (EI) *m*/*z*: 393 (M−1, 3%, C_14_H_17_Cl_2_N_2_O_3_PS), 256 (M−1, 7%, C_10_H_8_Cl_2_N_2_S), 161 (M, 23%, C_5_H_4_Cl_2_N_2_), 137 (M−1, 25%, C_4_H_11_O_3_P). Anal. Calcd. For C_14_H_17_Cl_2_N_2_O_3_PS (394.01): C, 42.55; H, 4.34; N, 7.09; P, 7.84. Found;C, 42.53; H, 4.28; N, 7.03; P, 7.79.

#### Diethyl (((3,5-dichloropyridin-2-yl)amino)(2-hydroxyphenyl)methyl)phosphonate, 3g

Yield (0.99 gm, 81%); 77 h; light beige solid; M.p.: 100–102 °C; TLC: Ethyl acetate: Petroleum ether 1:1); Rf: 0.23; IR: ν/cm^−1^: 3248 (NH), 3029 (CH–Arom.), 2925 (CH–Aliph), 1254 (P=O), 1086 (P–O–C), 761 (P–CH). ^1^H NMR (500 MHz, DMSO-*d6*): *δ* = 1.05–1.23 (m, 6H, 2 × CH_3_), 3.80–4.02 (m, 4H, 2 × CH_2_), 6.45 (s, 1H, CH–P), 6.91–8.48 (5H, s, 1H, OH, m, 4H, Ar–H),7.89 (s, 1H, H_pyridine_), 9.44 (s, 1H, H_pyridine_),10.22 (br, 1H, NH, exchangeable with D_2_O) ppm. ^13^C NMR (125 MHz, DMSO-*d6*): *δ* = 16.92 (2CH_3_), 66.17 (2CH_2_), 67.56 (CH-P), 154.68 (C=N pyridine), 166.47 (C–NH), 114.07–154.68 (Ar–C) ppm. MS (EI) *m*/*z*: 404 (M, 2%, C_16_H_19_Cl_2_N_2_O_4_P), 265 (M−3, 48%, C_12_H_10_Cl_2_N_2_O), 165(M+4, 14%, C_5_H_4_Cl_2_N_2_), 146 (M, 100%, C_5_H_3_Cl_2_N) 138 (M, 15%, C_4_H_11_O_3_P). Anal. Calcd. For C_16_H_19_Cl_2_N_2_O_4_P (404.05): C, 47.43; H, 4.73; N, 6.91; P, 7.64. Found;C, 47.40; H, 4.68; N, 6.85; P, 7.60.

### Antimicrobial activities

The produced compounds’ antimicrobial activity was evaluated using a panel of two gram-positive bacteria (*Bacillus subtilis* (*#*MTCC NO 441) and *Staphylococcus aureus* (#MTCC NO 96). as well as two Gram-negative bacteria (*Pseudomonas aeruginosa (#*MTCC NO 1688), *Escherichia coli*(#MTCC NO 452)). Two fungi [*Candida albicans*(#MTCC NO 183) and *Aspergillus flavus*(#MTCC NO 1344)] were used to assess the compounds' anti-fungal properties. Paper discs of Whatman filter paper with a standard size of 5 cm were made, dissolved in DMSO, and then sterilized in an autoclave. A solution of 1 mg/mL was prepared for each drug separately. Paper discs soaked in the desired concentration of the complex solution were aseptically placed in Petri dishes containing nutrient agar media (20 g + 3 g of beef extract + 5 g of peptone) seeded with bacterial and fungal strains. After 24 h of incubation at 36 °C, the inhibitory zones in the petri dishes were measured. There were three copies of every treatment. Using the same protocol as previously described, the antifungal Clotrimazole and the common standard antibiotic Ciprofloxacin were also tested for antibacterial activity at the same concentrations and solvent combinations[35].

### Antioxidant activity using DPPH and ABTS

With slight modifications, the DPPH and ABTS methods were used to test the new phosphonate’s free radical scavenging activity [36]. Briefly, one milliliter of a 0.1 mM DPPH in methanol solution and ABTS in distilled water were added to the newly synthesized compounds. For 30 min, the mixture was left in the dark. The synthetic compounds’ ability to scavenge free radicals was compared to that of L. Ascorbic acid. At 517 nm for DPPH and 734 nm for ABTS, optical density was recorded, and the concentration inhibition was computed.

The percentage of inhibition is equal to [(A control − A test)/A control] × 100, where A control represents the control absorbance and A test represents the test sample absorbance. It was established what sample concentration (IC_50_) produced 50% inhibition. Every experiment was conducted in triplicate, and the mean ± SE IC_50_ values were reported.

### Antitumor activity (in-vitro)

#### Cell lines

Mammary gland (MCF-7;# ATCC HTB-22), colorectal adenocarcinoma (Caco-2; *# *ATCC HTB-37), hepatocellular carcinoma (HepG-2; #ATCC HB-8065), and human lung fibroblast (WI-38; # ATCC CCL-75). The ATCC cell line was obtained via the Holding Company for Biological Products and Vaccines (VACSERA), Cairo, Egypt.

#### MTT assay

The MTT test was utilized to ascertain the phosphonate's inhibitory effects on cell growth utilizing the aforementioned cell lines. The basis of this colorimetric assay is the transformation of yellow tetrazolium bromide (MTT) by mitochondrial succinate dehydrogenase in living cells into a purple formazan derivative. 10% fetal bovine serum was added to RPMI-1640 media used to cultivate cell lines. At 37 °C in an incubator with 5% CO_2_, 100 units/mL of penicillin and 100 µg/mLof streptomycin were introduced as antibiotics. The cell lines were seeded at 1.0 × 10^4^ cells/well in a 96-well plate and kept at 37 °C for 24 h with 5% CO_2_. Following incubation, the cells were subjected to several concentrations of newly synthesized phosphonates and left for 48 h incubation. 20 µL of a 5 mg/mL MTT solution was added and incubated for 4 h after the drug treatment lasted for 48 h. To dissolve the purple formazan produced in each well, 100 µL of dimethyl sulfoxide (DMSO) was applied. Using a plate reader (EXL 800, USA), the colorimetric test is measured and recorded at the absorbance of 570 nm. (A570 of treated samples/A570 of untreated sample) X 100 was used to compute the relative cell viability as a percentage [37–39].

### Theory of calculations

All quantum chemistry calculations were performed using the Gaussian 09W program packages developed by Frisch and colleagues [40]. The structure of the molecules is optimized using DFT with Beck's three-parameter exchange functional and nonlocal correlation functional, Lee–Yang–Parr, B3LYP [41–43] utilizing the 6-311G++(d,p)basis set. HOMO (highest occupied molecular orbital), LUMO (lowest unoccupied molecular orbital), and MEP (m olecular electrostatic potential) charge density distributions were visualized using Gaussian view 05 software [44].

### Docking (in-silico)

The newly synthesized α-aminophosphonates were docked pertaining to dynamin-related protein 1 (DRP-1) (PDB: 1zws) (https://www.rcsb.org/structure/1ZWS) target mitochondrial fission and mitophagy protein. The target protein was taken out of the protein data bank, polarized then, native legend, and all water molecules were removed. To get more stable findings, a 3D drawing of the ligand molecules was made using Chemdraw Ultra 8.0. The ligand molecules and target proteins underwent energy reduction by the application of the MM2 force field prior to the docking procedure. Molecular docking was computed using the Molegro virtual docker programme (MVD) (http://www.molegro.com/mvd-product.php, 17–2–2021). Target protein and α-aminophosphonates interacted, as shown by the Discovery Studio® Visualizer 2016 program [45].

### ADMET pharmacokinetic characteristics

To study the pharmacokinetics and drug-likeness prediction of the newly synthesized compounds, the Swiss Institute of Bioinformatics’ online tool SwissADME (http://www.swissadme.ch/) was utilized. A 2D structural model of the chemical was converted into SMILES using SwissADME's SMILES generator. The SMILES data analysis was then used to determine the compound’s ADMET characteristics [46].

### Statistical analysis

Using GraphPad Prism software 6 (San Diego, CA) The values were derived based on the experimental data and expressed as mean ± SE.

## Results and discussion

### Chemistry

#### Synthesis and spectroscopic characterization

**Fig. 2:**
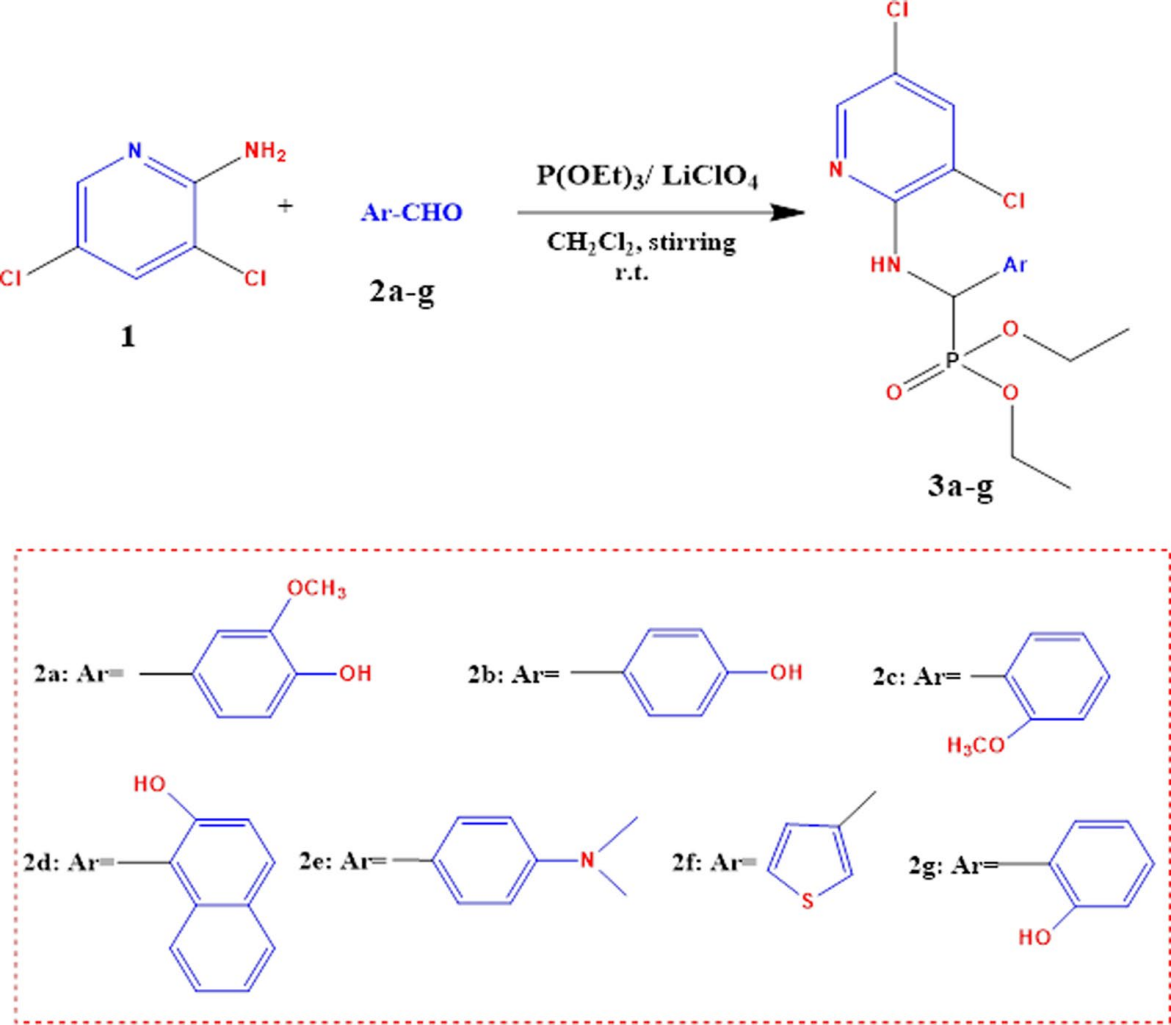
Synthetic pathway of α-aminophosphonates **3a**–**g**

3,5-dichloropyridin-2-amine **1**, different aromatic aldehydes **2a-g**, and triethyl phosphite were combined in a lithium per chlorate LiClO_4_ catalyst-aided process to create a sequence of Diethyl (((3,5-dichloropyridin-2-yl) amino)(Aryl) methyl) phosphonates (**3a**–**g**), Fig. 2.

FT-IR, ^1^H NMR, ^13^C-NMR spectroscopy, and Mass spectra, as well as corrected elemental analysis (Experimental portion), were used to confirm the structures of the studied α-aminophosphonates, **3a**–**g**.

The FT-IR spectra of compounds, **3a**–**g** were characterized by the following absorption bands, Fig.S1. A band is identified as the stretching vibration of the P=O group at 1289.74–1236.38 cm^−1^. υ (P–O–C) is responsible for a band that appeared at 1082.14–1022.98 cm^−1^, while υ (P–CH) is absorbed around 745.55–729.50 cm^−1^.At 3296.12–3138.99 cm^−1^, the CH aromatic stretching band is absorbed, and at 3167.72–2927.22 cm^−1^, the CH aliphatic bands are visible. At 637.21–625.74 cm^−1^, the stretching vibration C–Cl is absorbed. The bands between 1662.38 and 1565.18 cm^−1^ are thought to be the result of C=C stretching. NH/OH groups are finally absorbed between 3492.74 and 3386.05 cm^−1^.

The **3a**–**g** derivatives of α-aminophosphonate exhibit the following signals in their ^1^H-NMR (DMSO) spectra: The triplet signal at 0.98–1.23 ppm is caused by the 2 × CH_3_ aliphatic protons. A multiplet signal is generated by the 2 × CH_2_ aliphatic protons between 3.78 and 4.03 ppm. Signals between 5.18 and 6.46 ppm is produced by the CH aliphatic protons connected to the phosphonate group. In the 6.46–9.75 ppm range, the aromatic protons are reverberated as multiples. Finally, the NH group is responsible for the broad singlet signal around 10.25 ppm (Figs. S2–S9 supplementary materials). ^13^C-NMR (DMSO) analysis of the investigated substances shows the following signals: δ 63.81–72.06 (P–CH_aliph._), 13.56–27.72 (2 CH_3_), 57.45–66.17 (2 CH_2_), 144.17–155.04 (C=N_pyridine_), 154.73–166.47 (C-NH), and 99.80–154.68 ppm (C_Arom_.) (Figs. S10-–16 supplementary materials**)**. Morever, the full product’s mass spectrum revealed a peak at m/z that corresponded to the molecular ion (Figs. S17–S23, supplementary materials). Elemental microanalysis findings in agreement with corresponding theoretical values (Experimental part) were used to characterize the studied substances. Each spectrum confirms the structure of the synthesized compounds.

**Fig. 3:**
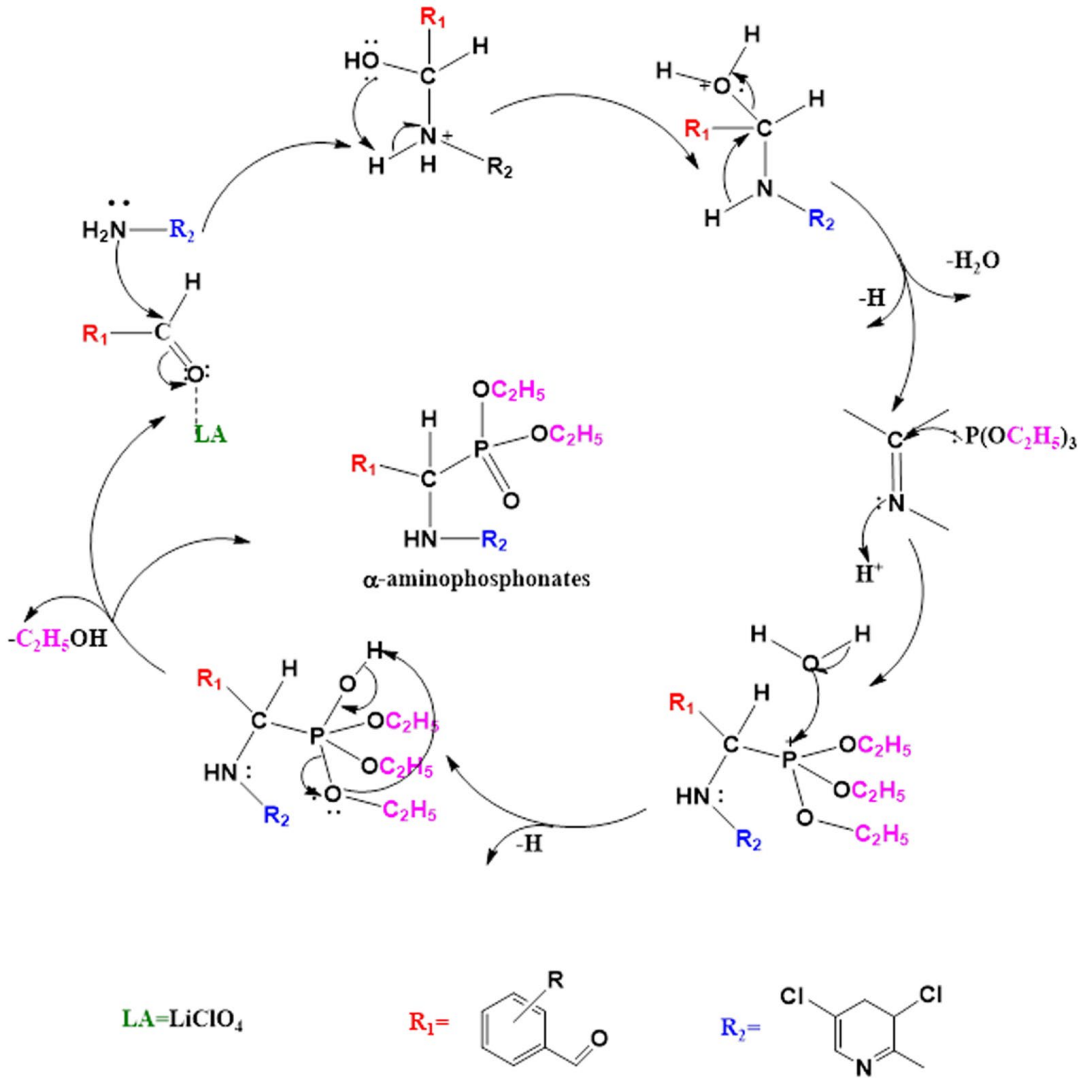
The probable mechanism for the LiClO_4_-Catalyzed Kabachnic-Fields Synthesis of Ethyl α-aminophosphonates **3a**–**g**

The proposed molecular pathway for the synthesis of α-aminophosphonate analogs was illustrated in Fig. 3, which involves two primary stages [47, 48]. (a) the in situ production of Schiff base through Lewis acid (Lithium perchlorate, LiClO_4_) catalyst-induced formyl group activation; By nucleophilically adding a (aminopyridine) nitrogen lone pair to the electrophilic carbon of the activated carbonyl group of -CHO, this promoted the condensation reaction between aminopyridine and aromatic aldehyde; (b) phosphorus atom (triethyl phosphite) nucleophilically attacking the electrophilic carbon of the imine moiety (> C=N–), which is followed by ethanol being released by phosphonium intermediates interacting with water to generate certain α-aminophosphonate analogs, Fig. 3.

### Biological investigation

#### Antimicrobial activity

**Fig. 4:**
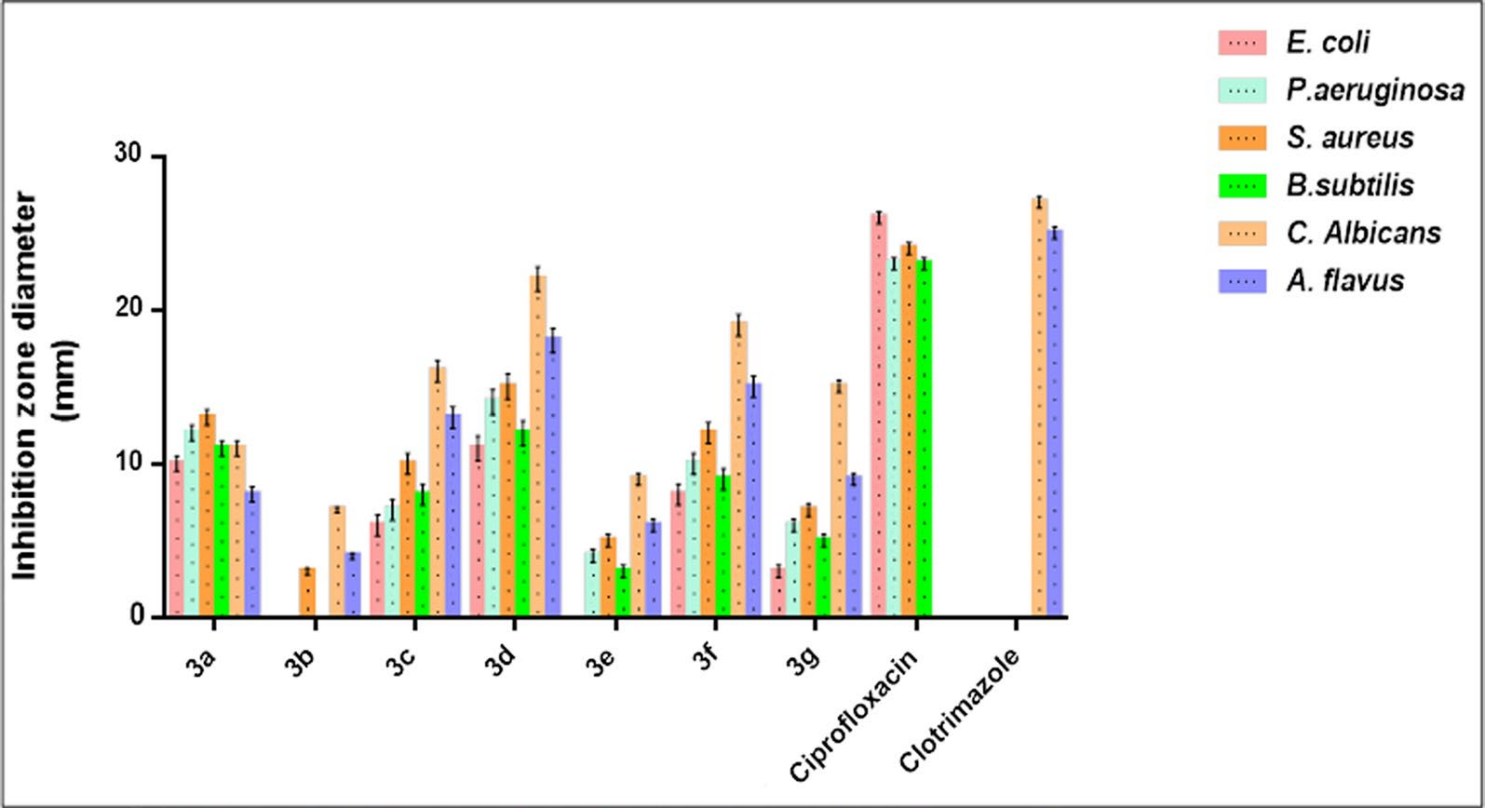
The antimicrobial activity of the synthesized compounds **3a**–**g** by disc diffusion method

Because of careless antibiotic use and insufficient infection control, resistant bacteria have grown to pose a serious threat to both public health and the world economy. Therefore, to stop the spread of antimicrobial resistance (AMR), it is imperative to carry out in-depth research and create a new class of antimicrobial compounds [45, 49]. Newly synthesized phosphonates were assessed for their *in-vitro* antibacterial activity using a standard agar well diffusion method against Gram-positive bacteria *S. aureus* and *B. subtilis*, Gram-negative bacteria *E. coli* and *P. aeruginosa*, and fungal *C. albicans* and *A. flavus*. Based on our findings, it was determined that compound **3d** had the strongest antibacterial and antifungal effects, preventing the growth of every germ under investigation compared with standard FDA approved drugs. This compound produced the biggest inhibitory zones with activity index equal to (42.3% for *E. coli*, 60.9% for *P. aeruginosa*, 62.5% for*S. aureus,* 52.2%for*B. subtilis*, 81.5% for *C. albicans*and 72% for*A. flavus*respectively). All other phosphonateshad from moderate as compounds **3a**, **3f** and **3c** to weak as compounds **3b**, **3e** and **3g** biocidal effect against bacterial and fungal strains, Fig. 4.

#### Antioxidant activity

**Fig. 5:**
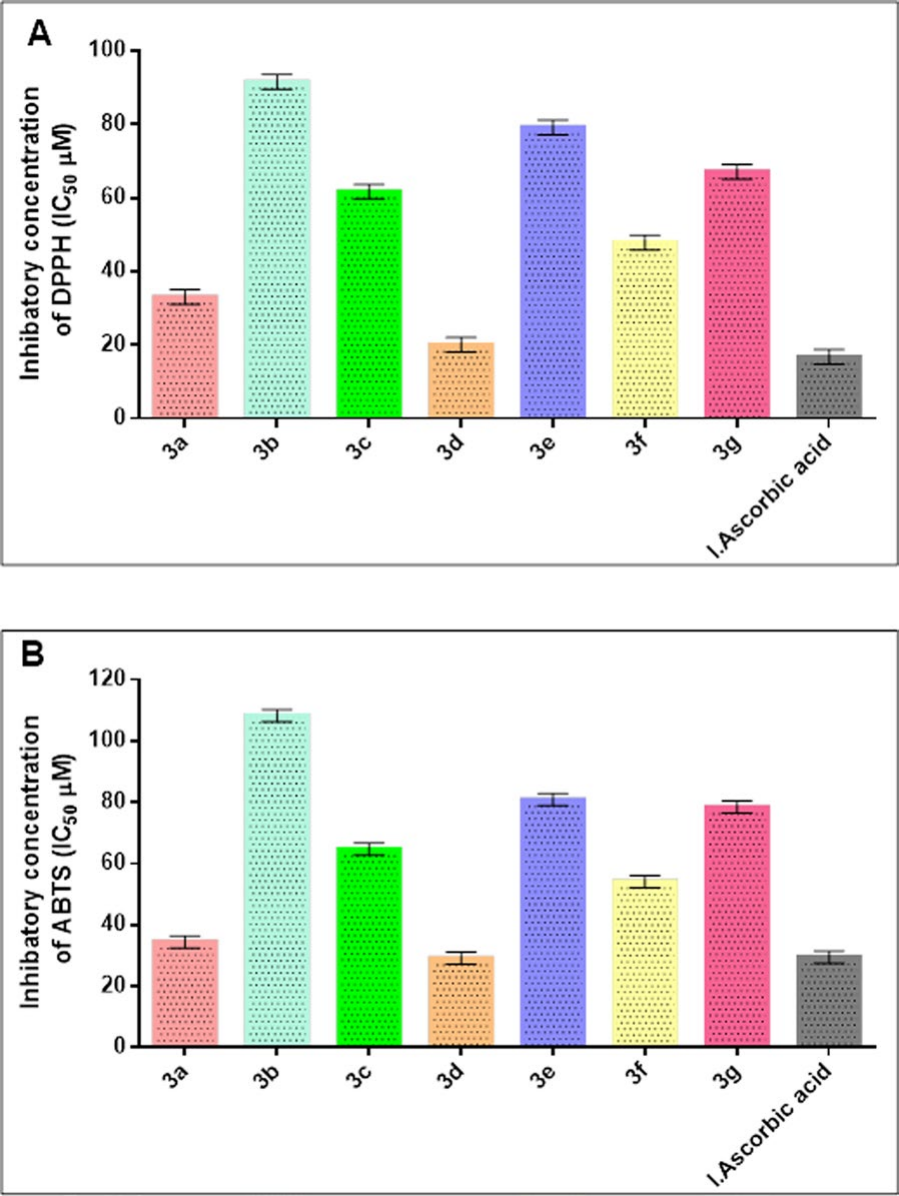
The antioxidant scavenging activity of all novel phosphonates **3a**–**g** using DPPH and ABTS

The stable DPPH and ABTS radical scavenging activity assays were utilized to determine the antioxidant potential of these novel phosphonates by measuring the change in absorbance generated, as shown in Fig. 5. The results demonstrated that the antioxidant activity increased with the concentration of these substances. Compound **3d** showed a higher DPPH IC_50_ value (20.04 ± 0.14 µM) compared to the conventional L-Ascorbic acid (IC_50_ = 16.81 ± 0.10 µM), according to our findings. The ABTS cation radical scavenging activity was examined using the decolorization test at various doses of phosphonates and compound **3d** exhibited the best IC_50_ with value equal to 29.14 ± 0.18 μM, compared to the standard L-Ascorbic acid which gave an IC_50_ value of 29.47 ± 0.17 μM. Furthermore, utilizing both DPPH and ABTS scavenging assays, compounds **3a** and **3f** shown a moderate impact, whereas compounds **3b**, **3c**, **3e**, and **3g** demonstrated a weak capacity to quench free radicals.

#### Molecular Docking (in-silico)

**Fig. 6:**
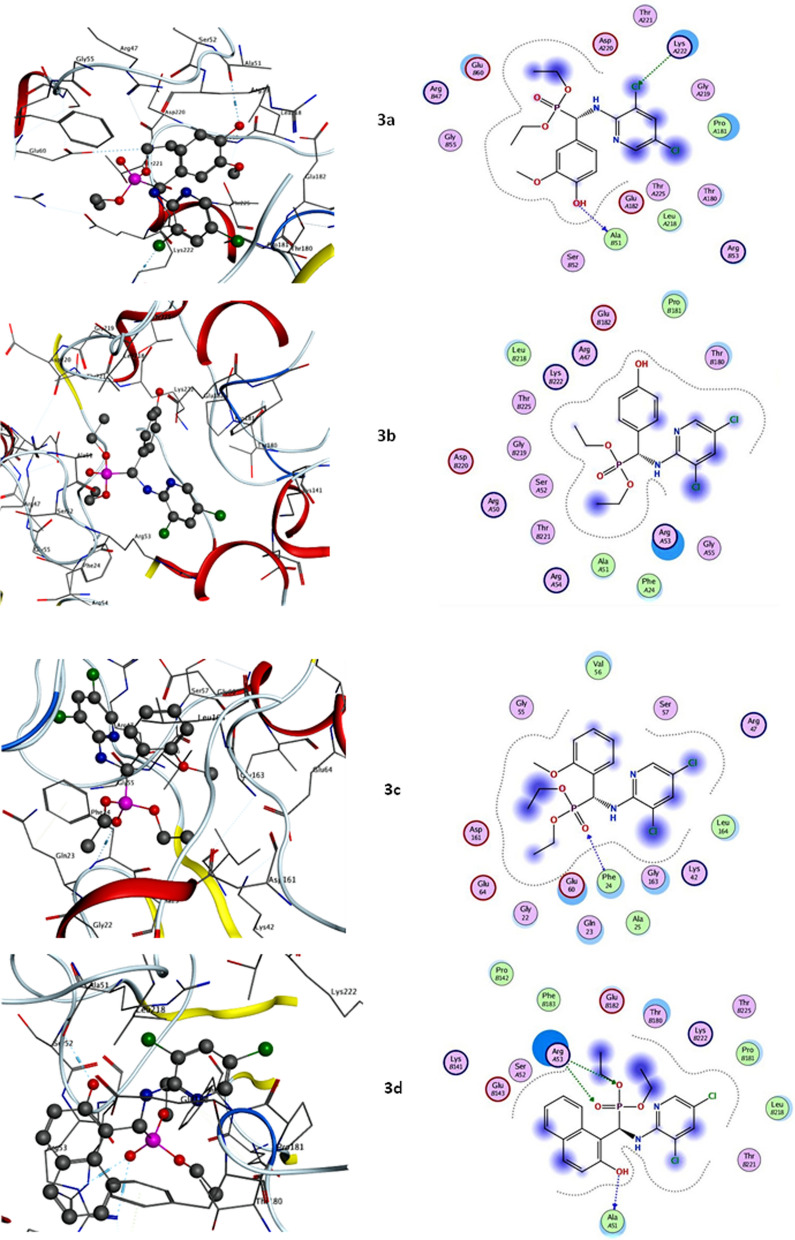

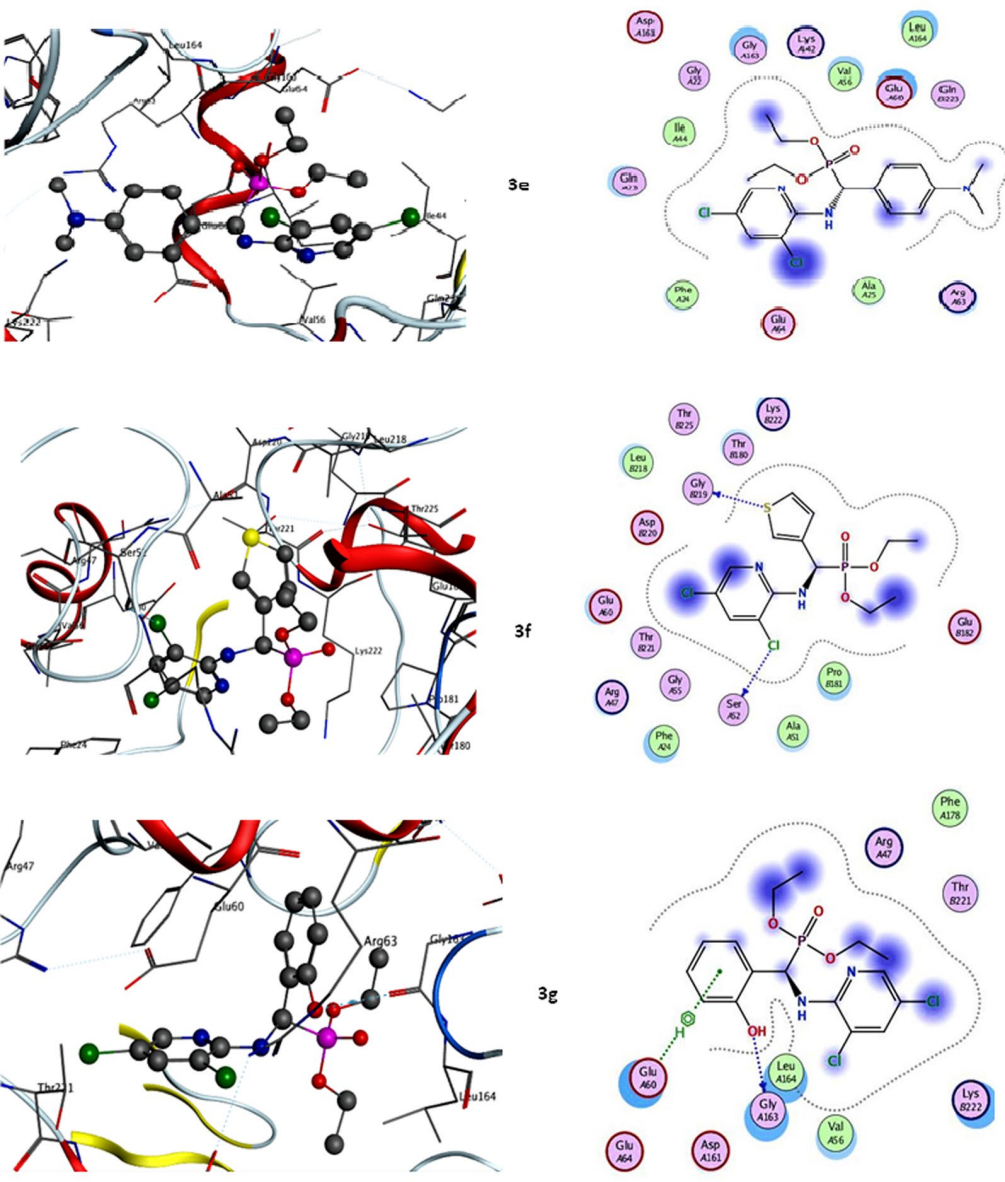
Molecular docking interactions between newly synthesized phosphonates **3a**–**g** and target DRP-1 mediated mitochondrial fission protein

Molecular docking has been widely used to identify novel drugs because it is an effective tool for fast and precisely estimating protein–ligand complex binding energies and biomolecular conformations. Herein, the novel phosphonate's ligands, **3a**–**g**, were docked into DRP-1 and are well-known, appealing therapeutic target proteins for the development of anticancer drugs. Mitochondrial division and fusion are engaged in the control of intrinsic apoptosis that is mitochondrial-dependent. This process was dependent on the release of mediators of cell death, such as cytochrome c, from the mitochondria and the permeabilization of the outer membrane [29]. Due to the function of ocular atrophy 1 protein (OPA1) in maintaining cristae, which reduces the release of cytochrome c produced by MOMP, mitochondrial fusion shields cells against apoptosis [50]. Numerous apoptotic models have been linked to mitochondrial fragmentation. Drp-1 plays a part in the permeabilization of the outer mitochondrial membrane (OMM) and cytochrome c release when it forms complexes with bcl-2-associated X protein (BAX) at mitochondrial fission sites [51]. Thus, Drp-1 plays a crucial role in many other aspects of cell biology, including apoptosis and cell death. All novel phosphonate’s interactions with target Drp-1 protein were described in Table 1; Fig. 6. Our results elucidated that compound **3d** exhibited the most binding energy against target Drp-1 proteinwith a value equal to − 9.54 kcal/mol. Furthermore, compounds **3a** and **3f** elucidated moderate inhibitory effect with binding energies equal to − 7.66 and − 7.16 K cal/mol respectively. On the other hand, compounds **3b**, **3c**, **3e,** and **3g** observed slightly weak binding energy equal to − 4.32, − 6.47, − 4.01, and − 6.20 kcal/mol respectively. Therefore, compound **3d** was strongly recommended to be used as an anticancer agent via its prospective inhibitory effect on Drp-1-mediated mitochondria fission.

**Table 1:** Calculated docking scores (kcal/mol) of all synthesized compounds **3a**–**g** with the target protein

Compounds	Dynamin-related protein 1 (DRP-1)	
	Docking score (ΔG bind)	Docked complex (amino acid–ligand) interactions
**3a**	−7.66	H-donor ALA51 H-acceptor LYS222 Electrostatic interactions PRO181 THR180 LEU218 GLU60 ARG47
**3b**	−4.32	Electrostatic interactions PRO181 THR180 LEU218 ARG53 PHE24 ALA51
**3c**	−6.47	H-donor PHE24 Electrostatic interactions VAL56 LEU164 GLY163 GLN23 GLU60 GLY22
**3d**	−9.54	H-donor ALA51 H-acceptor ARG53 Electrostatic interactions PRO142 PHE183 GLU182 THR180 PRO181 LEU218
**3e**	−4.01	Electrostatic interactions GLY163 VAL56 GLU60 LEU164 GLN23
**3f**	−7.16	H-donor GLY219 SER52 Electrostatic interactions LYS222 THR180 LEU218 GLU60 PRO181 ALA51 PHE24
**3g**	−6.20	H-donor GLY163 π-hydrogen GLU60 Electrostatic interactions LYS222 VAL56 LEU164 PHE178

#### Anti-tumor activity

**Fig. 7:**
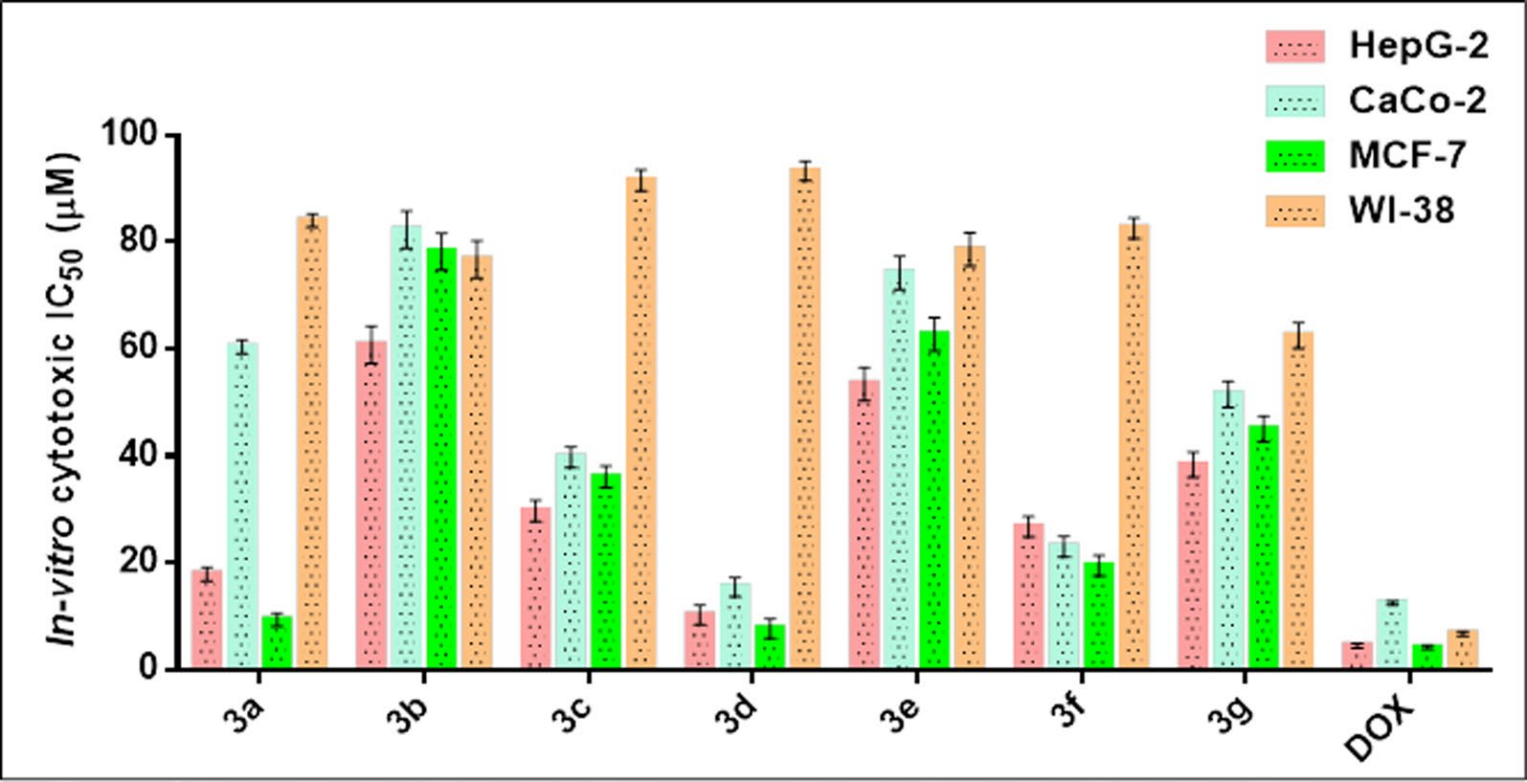
Antitumor/cytotoxic activity of compounds **3a**–**g**, against a panel of human tumor cells and a normal cell

In research on new anticancer agents, the most common experimental screening method after the theoretical study was testing against a group of different cancer cell lines [52, 53]. In this study, an MTT assay was done to determine the antitumor effect of phosphonates compounds on Caco-2, HepG-2, and MCF-7 proliferation, and the cytotoxicity limit on WI-38 normal cell line after 48 h, Fig. 7. Compound **3d** showed significant antitumor effects on Caco-2, HepG-2 and MCF-7 cancer cell lines with an IC_50_ equal to 15.47 ± 1.2, 10.23 ± 0.8, and 7.69 ± 0.5 μM, respectively. Also, compounds **3a** and **3f** showed remarkable antitumor effects on HepG-2 and MCF-7 cell lines with low effect on Caco-2 cells with IC_50_ values (17.92 ± 1.3,9.37 ± 0.8 μM) (26.79 ± 1.9,19.50 ± 1.3 μM) respectively. On the other hand, compounds **3c**, **3e**, and **3g** showed moderate to weak impact on all panels of cancer cell lines compared with the IC_50_ of DOX reference chemotherapeutic drug 12.49 ± 1.1, 4.50 ± 0.2and 4.176 ± 1.3 μM, respectively. Moreover, all newphosphonates showed lower cytotoxic effects on WI-38 normal cells compared with DOX which observed highly toxic effects on normal cells with IC_50_ equal to 6.72 ± 0.5 μM. This signifies that compounds **3d**, **3a**, and **3f**, in the same manner,were effective against proliferative cancer through inhibiting DRP-1 mediated mitochondrial fission and suppressing mitochondrial mitophagy that causesat the same time activation to BAX/ Cytochrome-C signaling proteins resulting in activating caspases enzymes and finally cause apoptosis without any toxic effects on normal cells. As a result of their potential inhibition of DRP-1 target protein, newly synthesized phosphonates could be exploited as therapeutic candidates for cancer therapy, according to docking and in-vitro studies.

#### ADMET in-silico drug-likeness and bioavailability features

**Fig. 8:**
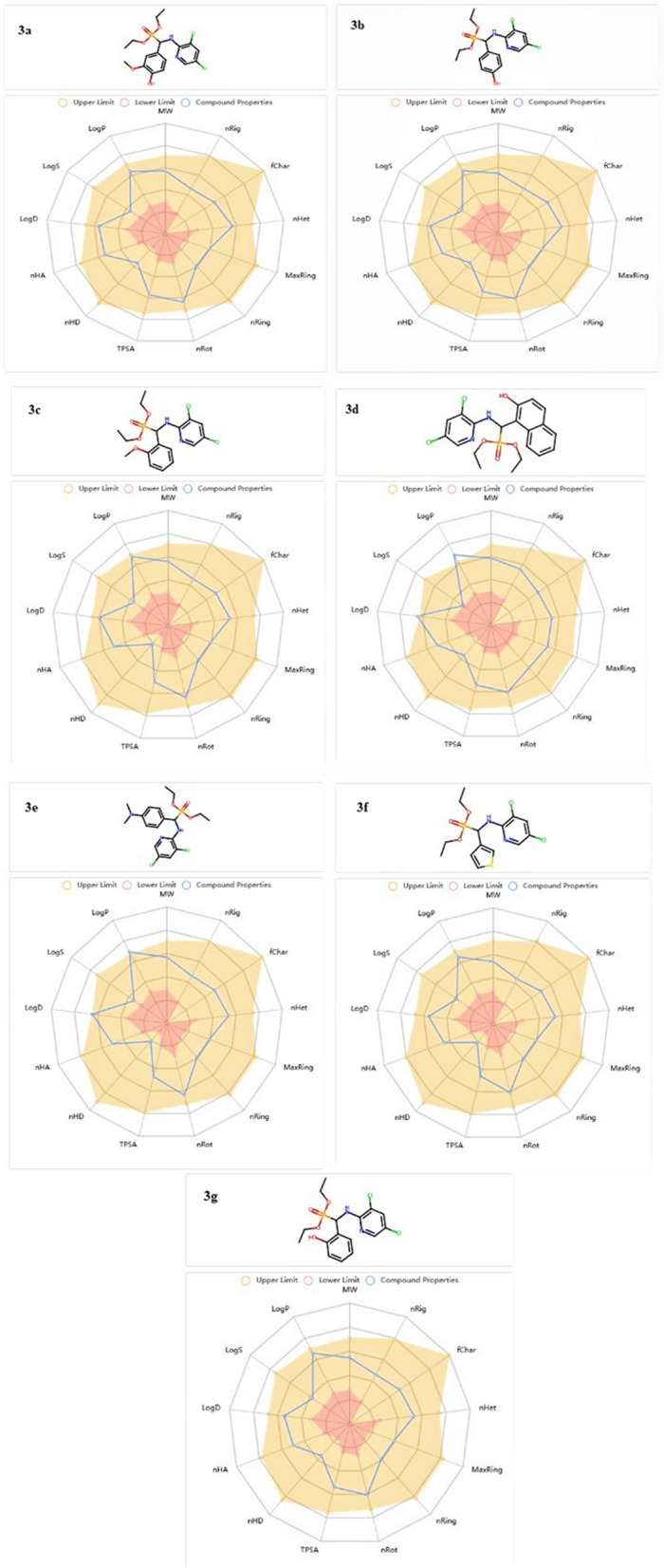
ADMET radar pharmacokinetics features of newly synthesized phosphonates

The ADMET has to certify the drug’s effectiveness as a top candidate against any illness [54]. Physio-chemical in-silico techniques were used to compute the donor hydrogen bond, drug similarity, and partition coefficient (cLogP). Furthermore, pharmacokinetic and bioavailability analyses have been performed to do these kinds of clinical studies on recently synthesized phosphonates. The topological polar surface area (TPSA) must be less than < 140 Å2 to have superior oral bioavailability. Our results showed that the phosphonate TPSA ranged from 60.45 to 89.91. Moreover, the results showed that phosphonates had good gastrointestinal absorptions and no BBB, indicating their CNS protection. Before being taken into consideration for development, the recently synthesized candidate needs to pass a toxicity risk assessment. Phosphonates did not exhibit any mutagenic harmful effects, according to the results of the AMES toxicity analysis. Remarkably, none of the substances proved carcinogenic, prompting an in-silico analysis, the results of which are shown in Table 2**, **Fig. 8. Our research indicates that the best-docked phosphonate **3d**, which also significantly inhibits the target DRP-1 protein, has appropriate physio-chemical, pharmacokinetic, and bioavailability in silico and seems to be a potentially useful new class of cancer therapies. It also showed no toxicity or carcinogenicity.

**Table 2:** ADMET properties of phosphonate compounds **3a**–**g**

	Molecular weight (g/mol)	Blood–brain barrier (BBB)	%Human intestinal absorption (HIA +)	TPSA _A_2	Log p	HBA	HBD	N rotatable	AMES toxicity	carcinogenicity
Acceptable ranges	≤ 500	NO	> 80% high < 30% low	≤ 140	< 5	2.0–20.0	0.0–6.0	**10 ≤**	Nontoxic	Noncarcinogenic
**3a**	434.060	NO	89.7	89.91	2.45	7	2	9	Nontoxic	Noncarcinogenic
**3b**	404.050	NO	90.5	80.68	2.52	6	2	8	Nontoxic	Noncarcinogenic
**3c**	418.060	NO	95.6	69.68	2.85	6	1	9	Nontoxic	Noncarcinogenic
**3d**	454.060	NO	99.7	80.68	3.7	6	2	8	Nontoxic	Noncarcinogenic
**3e**	431.090	NO	66.4	63.69	3.14	6	1	9	Nontoxic	Noncarcinogenic
**3f**	394.010	NO	89.5	60.45	2.59	5	1	8	Nontoxic	Noncarcinogenic
**3g**	404.050	NO	92.6	80.68	2.66	6	2	8	Nontoxic	Noncarcinogenic

#### Structure–activity relationship analysis (SAR) of α-aminophosphonates

**Fig. 9:**
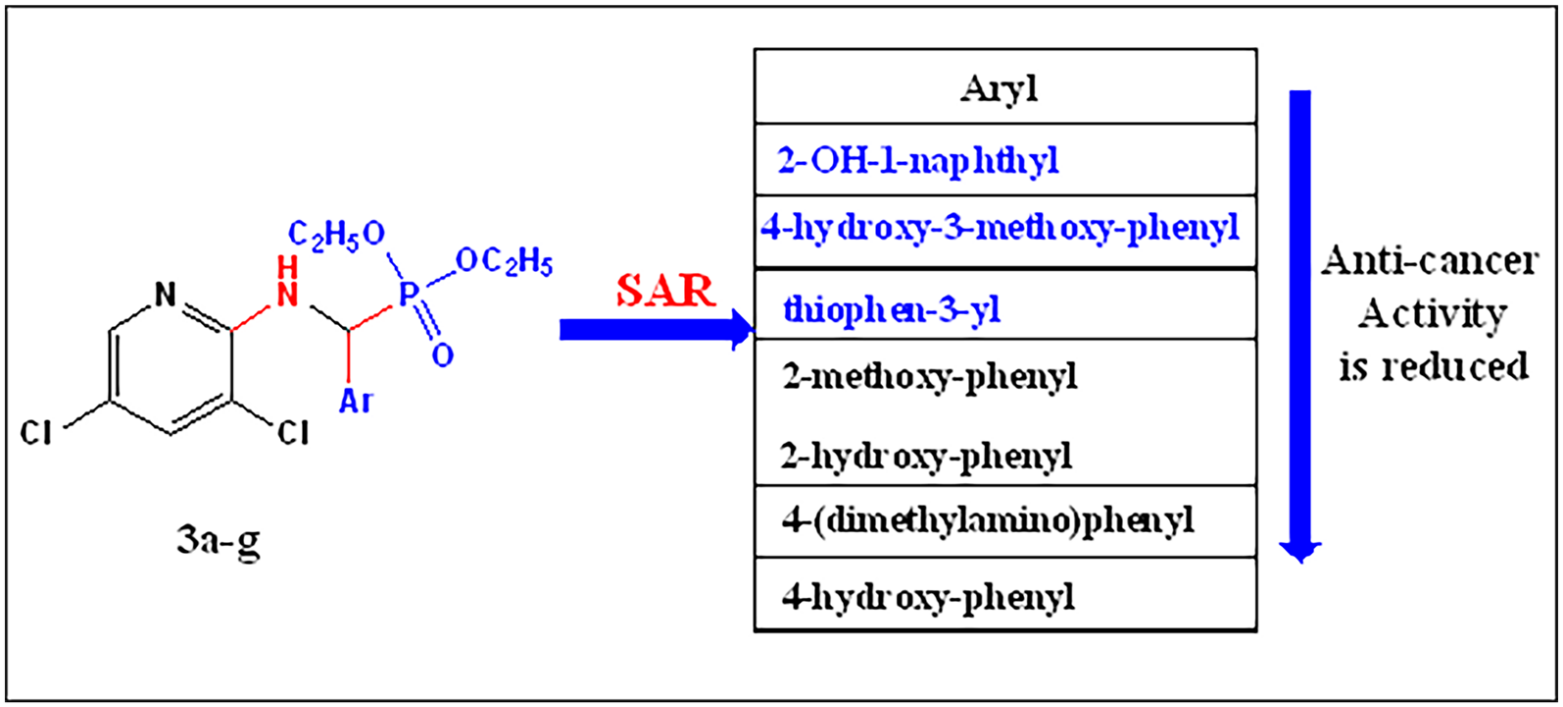
Structure–activity relationship analysis (SAR) of Diethylaminophosphonates **3a**–**g**

To our knowledge, numerous research examining the relationship between α-aminophosphonates' structure and activity (SAR relationship) have demonstrated their anti-cancer properties.Regarding the structural surface area ratio (SAR) of the molecule, Fig. 9, a consistent relationship was observed between the anti-cancer activity and the electrical characteristics and/or lipophilicity of the substitute Aromatic (Ar) groups. The sequence of the investigated compounds’ action against cancer cells is indicated in Table 3 with regard to the nature of substituent group Ar: (2-hydroxy-1-naphthyl>4-hydroxy-3-methoxy-phenyl>thiophen-3-yl>2-methoxy-phenyl>2-hydroxy-phenyl>4-(dimethylamino)phenyl>4-hydroxy-phenyl), Fig. 9. From the previous configuration, the addition of a naphthyl group (a more π-conjugated ring system) to the diphenyl phosphonates increases their anticancer activity against HepG-2, MCF-7, and Caco-2, with IC_50_ values of 10.23, 7.69, and 15.47, respectively. It might originate from naphthalene's significant medicinal advantages[55].Also, the presence of the 4-hydroxy-3-methoxy-phenyl group (i.e. vanillin) in the phosphonate skeleton enhances the biological activity [56].

**Table 3:** Structure–activity relationship (SAR) of α-aminophosphonate compounds **3a–g**
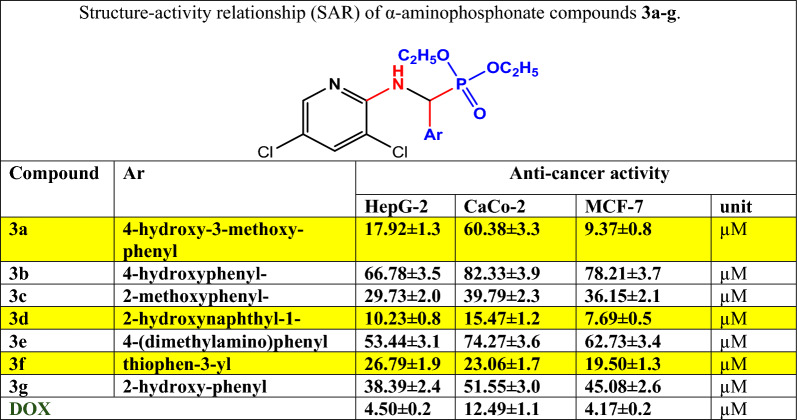

Compound	Ar	Anti-cancer activity			
		HepG-2	CaCo-2	MCF-7	Unit
**3a**	4-Hydroxy-3-methoxy- phenyl	17.92 ± 1.3	3.3 ± 60.38	0.8 ± 9.37	µM
**3b**	4-Hydroxyphenyl-	66.78 ± 3.5	3.9 ± 82.33	3.7 ± 78.21	µM
**3c**	2-Methoxyphenyl-	2.0 ± 29.73 ± 2.0	2.3 ± 39.79	2.1 ± 36.15	µM
**3d**	2-Hydroxynaphthyl-1-	0.8 ± 10.23	1.2 ± 15.47	0.5 ± 7.69	µM
**3e**	4-(dimethylamino)phenyl	3.1 ± 53.44	3.6 ± 74.27	3.4 ± 62.73	µM
**3f**	Thiophen-3-yl	1.9 ± 26.79	1.7 ± 23.06	1.3 ± 19.50	µM
**3g**	2-Hydroxy-phenyl	2.4 ± 38.39	3.0 ± 51.55	2.6 ± 45.08	µM
**DOX**		6.72 ± 0.5	4.50 ± 0.2	12.49 ± 1.1	μM

### Computational details

#### Quantum chemical calculations

**Fig. 10:**
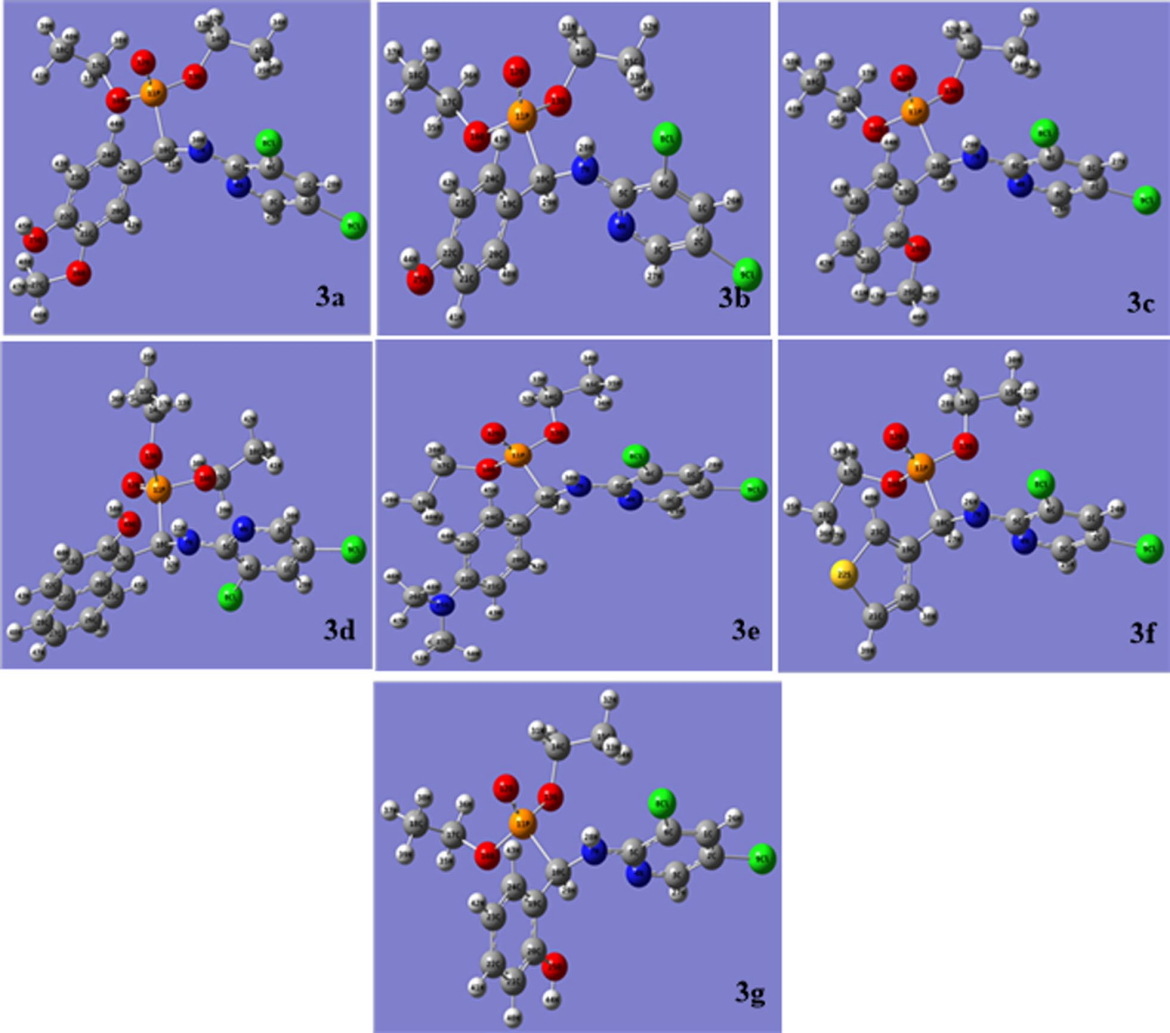
Optimized α-aminophosphonate structures **3a**–**g**

Many different molecule properties, such as reactivity, shape, and binding locations, as well as molecular fragments and substituents, can be defined using quantum chemistry techniques and molecular modeling processes. Quantum chemical computations were utilized to examine the correlation between the activity of α-aminophosphonates and structural variables. The density DFT technique was used in the computational study through Beck’s three-parameter exchange functional (B3LYP) with 6-311G++(d, p) basis set implemented in the Gaussian 09 program package to optimize the molecular structures of the compounds under investigation. Figure 10 shows the optimized chemical systems with the lowest energy discovered by calculations of the studied substances.

Quantum chemical calculations revealed that compound **3d**, which contains a 2-naphthol substituent in the aryl aldehyde moiety of the α-aminophosphonates, exhibited more powerful biological activity in comparison to compound, **3g**, which has a 2-OH-phenyl moiety and the remaining phosphonates. To enhance its capacity to take in electrons from the target protein, compound **3d** has the highest LUMO energy (− 1.6964 eV), Table 4**,** and is anticipated to respond as an electrophile (electron acceptor). Furthermore, a simple method for examining donor–acceptor behavior in a chemical system is provided by hardness (η) and softness (σ). A soft molecule has a small energy gap, whereas a hard molecule has a big energy gap [57]. As a result, soft molecules will polarize more readily than hard molecules. The results shown in Table 4 demonstrate that the **3d** phosphonate exhibited greater softness (0.4454 eV) than **3g** (0.4233 eV). One measure of a molecule’s electron-absorbing capacity is the global electrophilicity ω index [58]. When compared to other phosphonates, compound **3d** exhibits superior electrophilicity characteristics.The observed reactivity index by energy stabilization is the largest range of electronic loads (ΔN_max_) obtained from the environment (donor) through the inhibitor (acceptor). Based on the calculation, compound **3d** has the highest ΔN_max_ (1.7555e), Table 4. This indicates that it allows for charge transfer and an alternation of electron density between the compound and protein, leading to an excellent fit with the experimental data regarding the growth of biological activity.After analyzing the conversation, the computational calculations indicated that **3d**, α-aminophosphonate, boosts reactivity more than the other phosphonates, which may be more advantageous for the enzyme's reactivity and agrees with the experimental data appropriately, Table 4.

**Table 4:** Calculated parameters received from DFT/B3LYP/6-311G++(d, p) of the α-aminophosphonates **3a**–**g**

Compound	EHOMO (eV)	ELUMO (eV)	ΔE (ELUMO-EHOMO) (eV)	Dipole moment DM (D)	Ionization potential IP (eV)	Electron affinity EA (eV)	Hardness η (eV)	Softness σ (eV^−1^)	Chemical potential µ (eV)	Electronegativity χ (eV)	Electroph- ilicity ω	ΔNmax
**3a**	− 9.80481	− 1.35132	8.453494	3.3047	9.804812	1.351318	4.226747	0.236589	− 5.57806	5.578065	3.680704	1.319706
**3b**	− 9.75991	− 1.72956	8.030356	10.2402	9.759913	1.729557	4.015178	0.249055	− 5.74473	5.744735	4.109653	1.430755
**3c**	− 9.3512	− 1.2988	8.052397	3.9811	9.351198	1.2988	4.026199	0.248373	− 5.325	5.324999	3.521388	1.322587
**3d**	− 9.39855	− 1.30914	8.089405	3.4263	9.398545	1.30914	4.044702	0.247237	− 5.35384	5.353843	3.543355	1.323668
**3e**	− 8.83908	− 1.30315	7.535925	6.8801	8.839079	1.303154	3.767963	0.265395	− 5.07112	5.071117	3.412484	1.345851
**3f**	− 9.73161	− 1.31132	8.420296	6.0777	9.731613	1.311317	4.210148	0.237521	− 5.52147	5.521465	3.620607	1.311466
**3g**	− 8.99582	− 1.31213	7.683683	5.4237	8.995817	1.312134	3.841842	0.260292	− 5.15398	5.153975	3.457126	1.341538

#### The frontier molecular orbitals (FMOs)

**Fig. 11:**
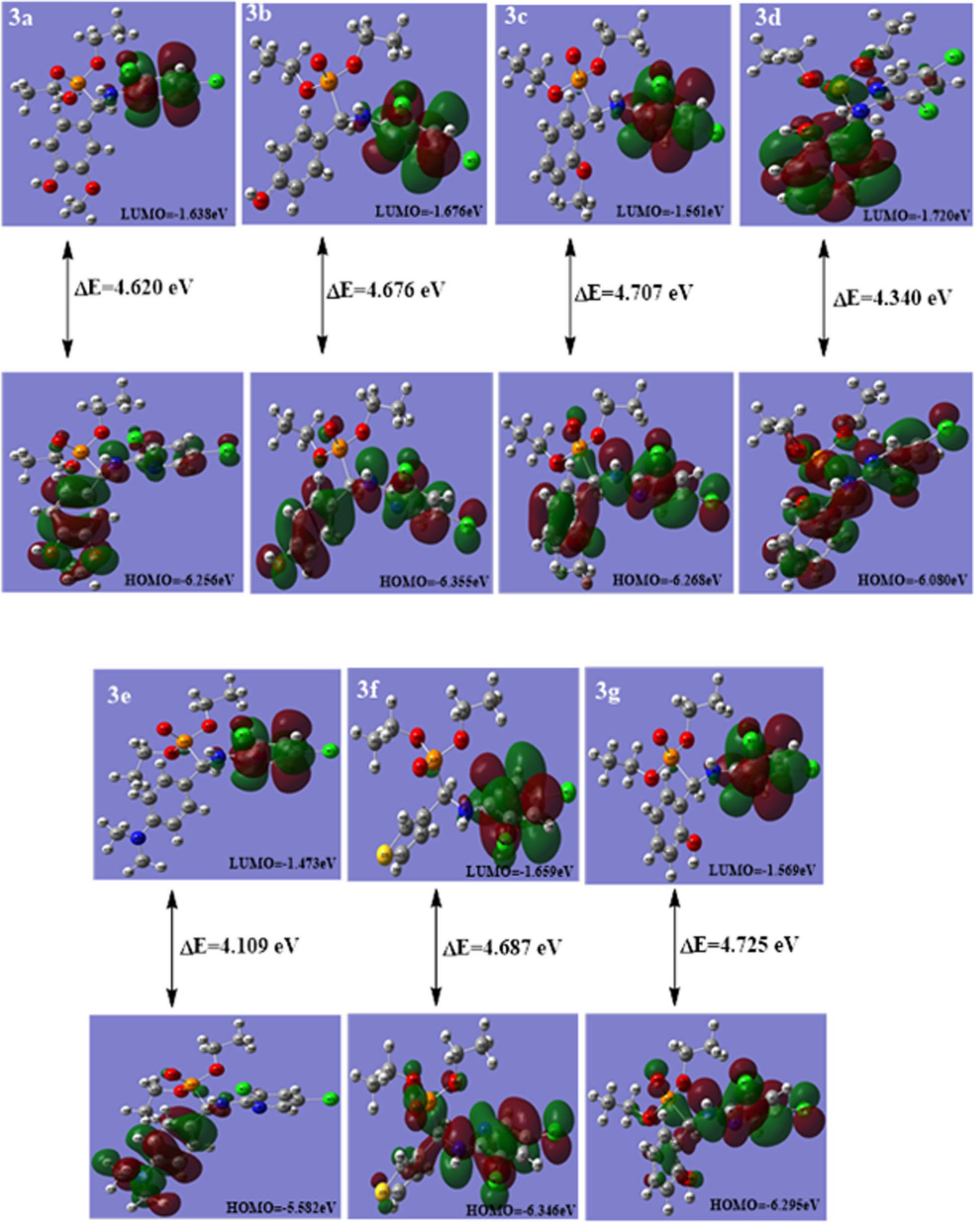
The calculated HOMO, LUMO of compounds **3a**–**g**

The highest energy of matched electrons (E_HOMO_), and the lowest energy of unmatched electrons, (E_LUMO_), can be used to assess the characteristics of excitation and the electron's carrying capacity [59–61].They are essential to the chemical stability of the molecule [62]. The FMOs allow researchers to predict molecular interactions. Whereas the LUMO is mostly an electron acceptor, the HOMO is primarily an electron giver [63].The distinction between HOMO and LUMO determines the molecules’ chemical stability and reactivity. As seen in Fig. 11, the HOMO–LUMO orbitals, their distributions, and energy levels were estimated at the B3LYP/6-311G++(d, p) level. for every synthesized α-aminophosphonate molecule.For compound **3d**, other than the ethyl ester groups, the HOMO distributes charges throughout the molecule, which can be interacted as a nucleophile (hydrogen bond donor) with the biological target, while its LUMO is selectively delocalized at the naphthyl group,which functions as an electrophile to interacte with the biological target (hydrogen bond acceptor).For compound **3e**, the HOMO distributes charges over the substituted phenyl moiety containing N(CH_3_)_2_ substituent, while its LUMO distributes charges over the di chloro pyridine moiety. The HOMO of the remaining phosphonate compounds distributes charges over the molecules except for the ethyl ester group, while the LUMO distributes charges over the di chloro pyridine moiety only Fig. 11.

#### Molecular electrostatic potential

**Fig. 12:**
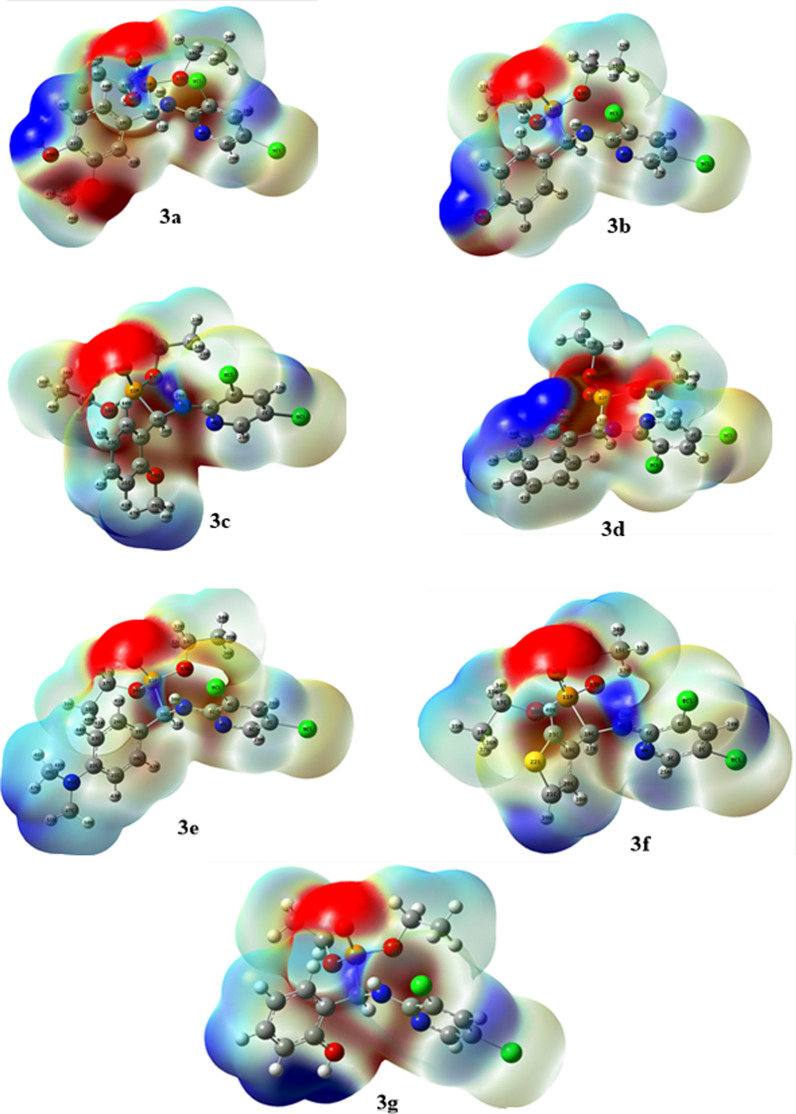
The calculated MEP of compounds **3a**–**g**

Molecular electrostatic potential (MEP) is a three-dimensional representation of a molecule’s charge distributions. Every molecule's properties, including its dipole moment, electronegativity, partial charges, and chemical reactivity, can be connected through the use of molecular electrostatic potential [64]. Analyzing phenomena like electrophilic and nucleophilic sites, hydrogen bonding interactions, solvent effects, and more can be done with a molecular electrostatic [65, 66]. Different colors designate the zones of positive, negative, and neutral potentials. The parts that are red and yellow are linked to electrophilic reactivity and correspond to the area of high electron density [67]. White represents a zone of positive electrostatic potential, whereas blue represents low electron density and nucleophilic reactivity. On the other hand, green coloursrepresent regions of zero potential [68]. To tighten the reactive zone (attacks by electrophile and nucleophile sites) for phosphonates, molecular electrostatic potential is computed. The potential zones that are neutral, negative, and positive are represented by different colors.The red and yellow zones, which correspond to the region of high electron density, are associated with electrophilic reactivity. A zone of positive electrostatic potential is represented by white, whereas a zone of low electron density and nucleophilic reactivity is represented by blue.Green, on the other hand, denotes areas with zero potential. These regions of varying electrostatic potential can offer helpful information about many kinds of intermolecular interactions and help in predicting the molecule's chemical behavior. The MEP graphs for the produced compounds, **3a**–**g** are shown in Fig. 12.

The phosphonate group’s oxygen atoms contained the electron-rich regions. These locations designate the molecular segments that are appropriate for electrophilic reactions. By examining the MEP plots, one can determine the electrostatic forces that interact between the investigated compounds and the organic target, Fig. 12.

#### Vibrational spectra

**Fig. 13:**
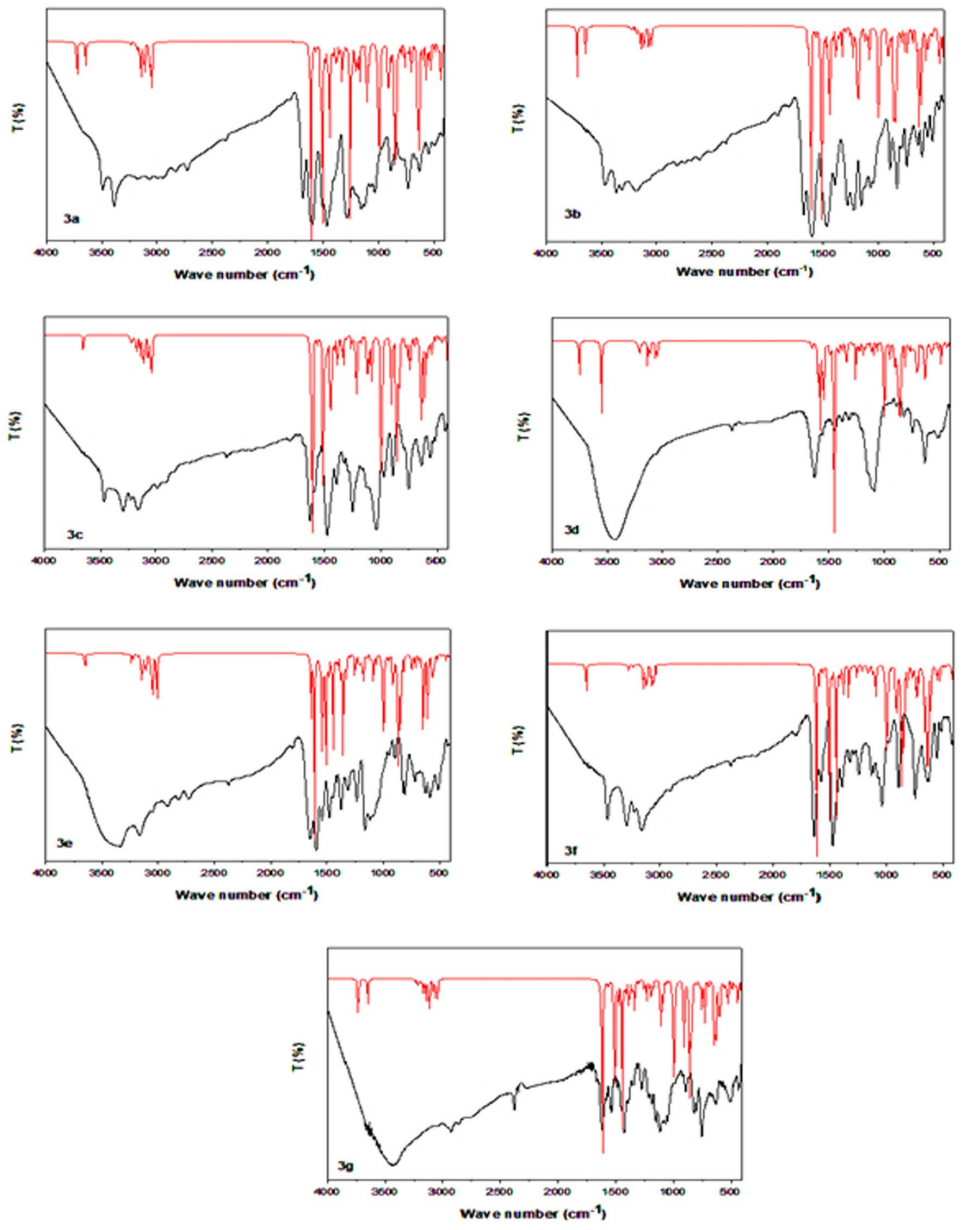
Comparison of the computed (red color) and experimental (black color) FT-IR spectra of the α-aminophosphonates **3a**–**g**

**Fig. 14:**
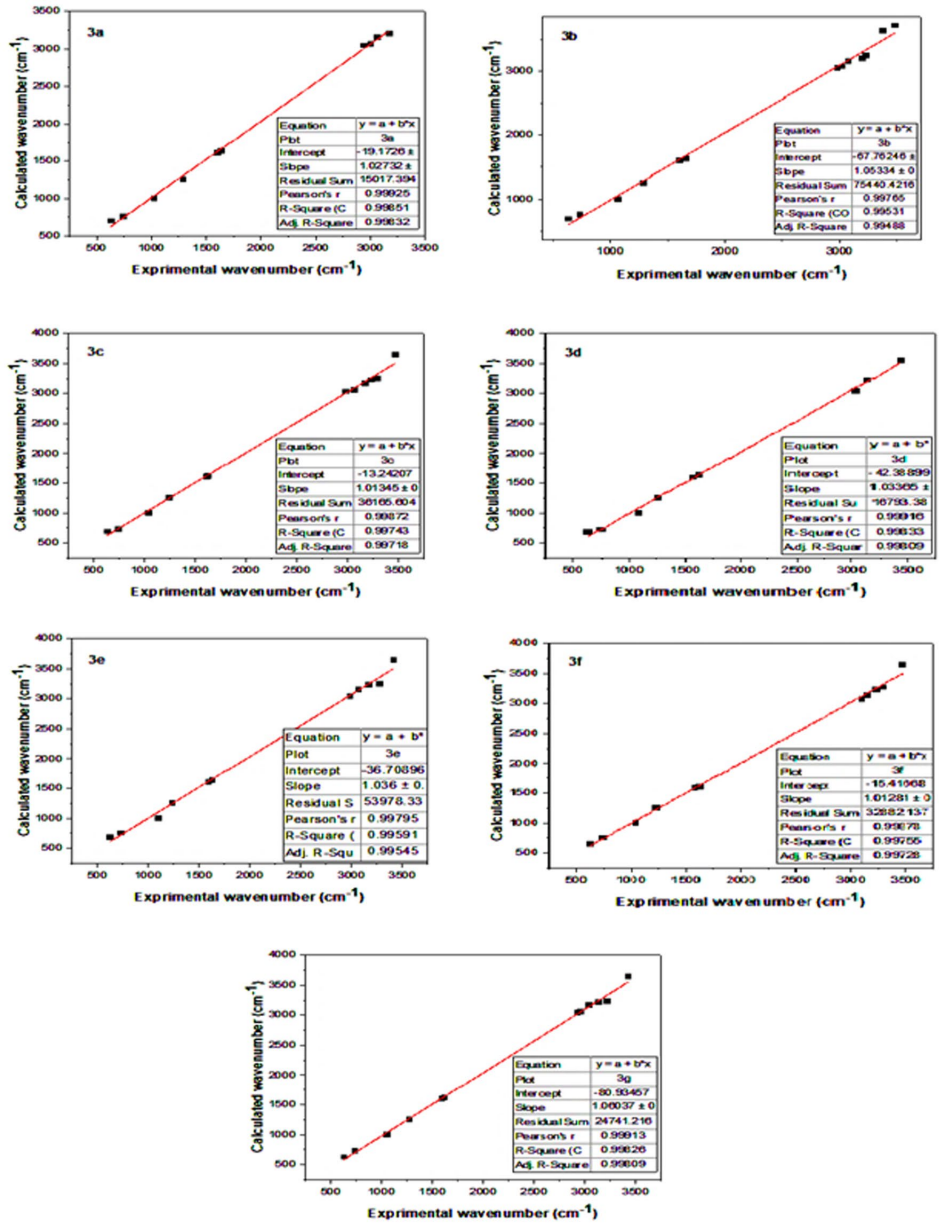
Correlation charts between the experimental and computed frequencies of the chemicals under investigation

Table 5 depicts the correlation between scaled wavenumbers and experimental wavenumbers of the substances under examination using DFT/B3LYP/6-311G++(d, p) method. Figure 13 presents a visual representation of the observed and computed infrared spectra. The appropriate wavenumber assignments to a given vibration are determined by comparing the calculated and experimental wavenumbers. The difference in wave number values between the observed and projected values can be explained by contrasting the solid phase data with theoretical estimates for the gaseous phase [69]. The symmetric stretching vibrations of the N–H group are responsible for the broadband (3492.74–3375.37) cm^−1^ that was seen in the experiment, while the calculated wide band is (3645.94–3550.62) cm^−1^. The typical aromatic C-H stretching is responsible for the band that is seen at (3296.12–3138.99) cm^−1^, whereas the calculated band is located at (3247.80–3196.07) cm^−1^.The observed CH aliphatic stretching band is (3150.30–2927.22) cm^−1^, while the calculated one is (3214.16–3013.40) cm^−1^. Also, the stretching vibration of the P = O group may be crucial for studying the α-aminophosphonates that are absorbed in (1289.74–1236.38) cm^−1^ (experimental) and at (1257.23–1252.38) cm^−1^ (calculated). The measured and calculated wave-wide variation υ (P–O–C) were observed at (1103.17–1022.98) cm^−1^ and (999.90–996.88) cm^−1^, respectively. Furthermore, one of the most significant phosphonate characteristic bands is υ (P–CH), which is observed at (745.55–729.50) cm^−1^ in experiments and (761.58–723.44) cm^−1^ in calculations. The band was ultimately found to be caused by υ (C–Cl) at (637.21–625.74) cm^−1^ (experimental) and (702.00–652.27) cm^−1^ (calculated). The calculated and experimental findings exhibit a high degree of accuracy, as evidenced by the linear correlation coefficient (R) value of 0.998, Fig. 14**.**

**Table 5:** Comparison of the experimental and calculated vibrational frequencies (cm^−1^) of α-aminophosphonates **3a**–**g**

Band assignment (cm^−1^)	NH/OH stretch		CH stretch aromatic		CH stretch aliphatic		C=C stretch		C=N stretch		P=O stretch		P–O–C stretch		P–CH stretch		C–Cl	
Compound	Exp	Cal	Exp	Cal	Exp	Cal	Exp	Cal	Exp	Cal	Exp	Cal	Exp	Cal	Exp	Cal	Exp	Cal
**3a**	3492, 3392	3723, 3646	3173, 3067	3204, 3155	3002, 2938	3070, 3048	1631	1630	1602	1608	1290	1257	1023	997	746	760	628	702
**3b**	3482, 3375	3720, 3641	3226, 3194, 3077	3247, 3199, 3158	3023, 2981	3074, 3047	1662	1636	1602	1608	1288	1252	1069	997	733	762	637	691
**3c**	3469	3645	3296, 3232, 3168	3247, 3227, 3173	3061, 2986	3063, 3037	1621	1626	1610	1606	1243	1253	1040	997	746	730	634	693
**3d**	3442	3551	3139	3227	3043	3050	1629	1634	1565	1595	1269	1256	1082	1000	745	723	636	690
**3e**	3416	3652	3279, 3170	3247, 3232	3070, 2988	3153, 3045	1632	1639	1595	1609	1240	1255	1103	999	730	756	629	685
**3f**	3469	3649	3296, 3232	3275, 3232	3150, 3096	3142, 3072	1628	1609	1583	1583	1236	1253	1036	998	744	757	626	652
**3g**	3429	3647	3215, 3141	3232, 3218	3045, 2959, 2927	3168, 3063, 3047	1617	1615	1596	1607	1274	1253	1057	997	740	728	631	631

#### NMR spectral analysis

**Fig. 15:**
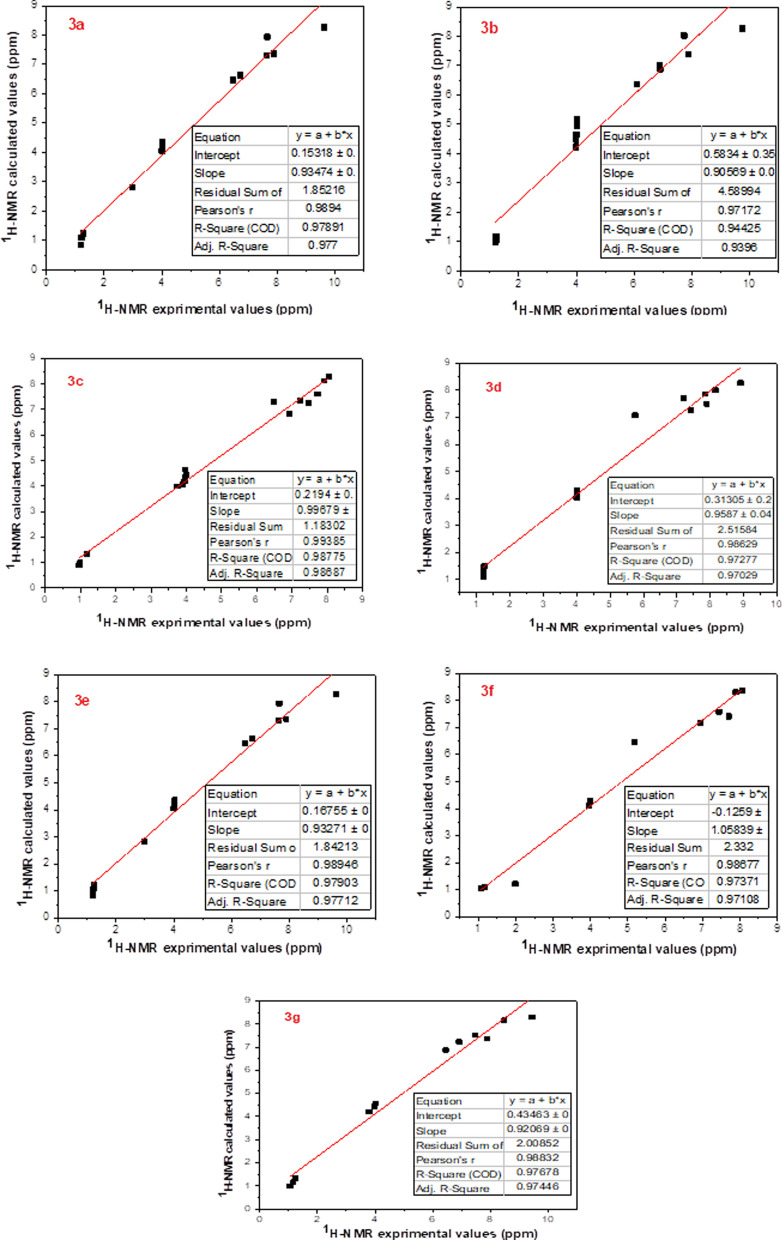
Comparison between the theoretical ^1^H-NMR chemical shift values and the experimental results for the α-aminophosphonates **3a**–**g**, as measured by the B3LYP/6311G (DMSO) method

**Fig. 16:**
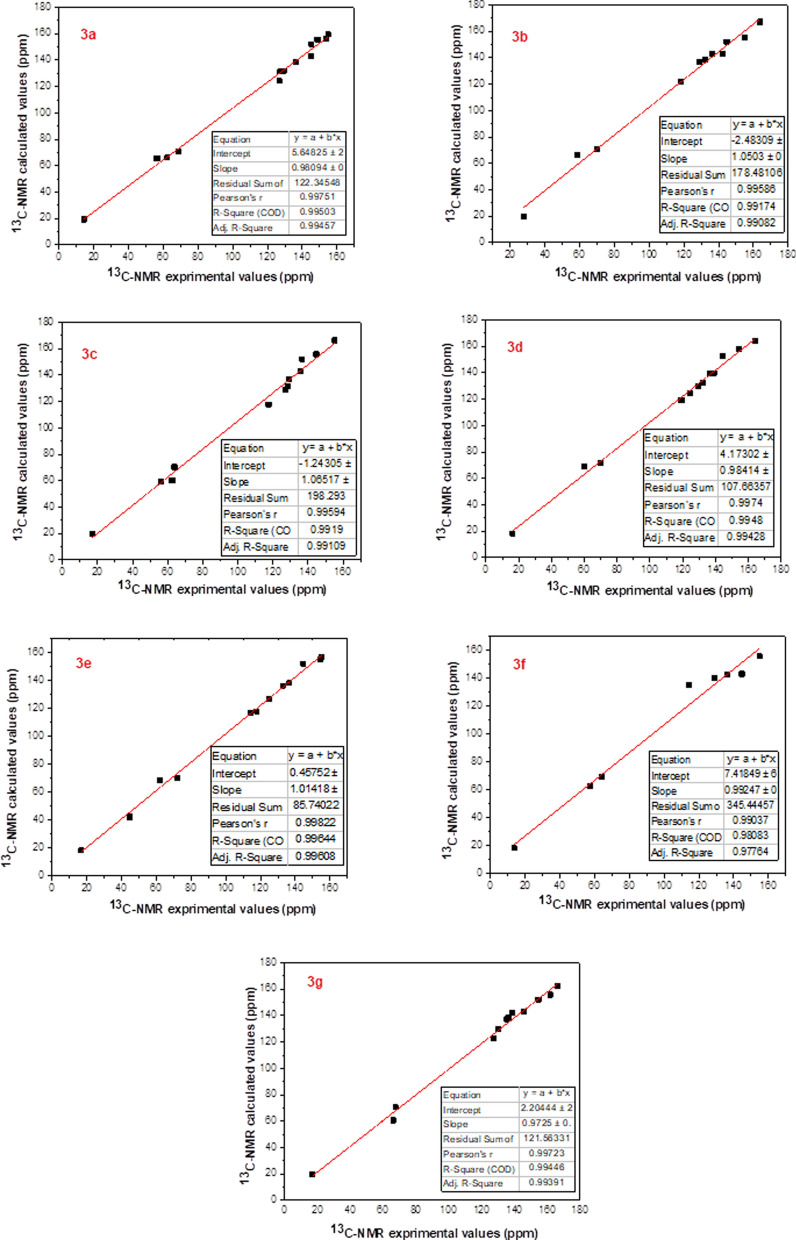
Comparison of experimental data with theoretical ^13^C-NMR chemical shift values obtained by B3LYP/6311G (DMSO) method for the α-aminophosphonates **3a**–**g**

Modern chemistry relies heavily on Nuclear Magnetic Resonance (NMR) spectroscopy as a crucial research tool for precise molecular geometry predictions [70–72]. Several chemical areas have developed applications for computational NMR [73]. Part of theoretical research involves the active computation of some crucial NMR parameters, including chemical shifts, shielding constants, and nuclear spin–spin couplings [74]. GIAO (Gaussian with Gauge In-dependent Atomic Orbital) was used to obtain the theoretical chemical shifts of α-aminophosphonates [75]. based on the improved B3LYP/GIAO/6-311G technique in DMSO solvent. Experimental and computed ^1^H-NMR and ^13^C-NMR spectrum data are summarized in Tables 6 and 7, respectively. Figures 15 and 16 show a comparison of the theoretically and experimentally obtained ^1^H-NMR and ^13^C-NMR isotropic shift values, indicating a linear association with a high correlation coefficient.The results demonstrated a high degree of agreement between the GIAO-NMR method and the experimental NMR chemical shift values.

**Table 6:** Theoretical and experimental ^1^H-NMR chemical shifts (ppm) of **3a**–**g** compounds computed at the level of the DFT/B3LYP/6-311G theory

			Chemical shifts, δ (ppm)					
** 3a**			** 3b**			** 3c**		
	Experimental ^a^	Theoretical ^b^		Experimental ^a^	Theoretical ^b^		Experimental ^a^	Theoretical ^b^
**H28, H29 pyridine**	7.89 (s), 9.74 (s)	7.38, 8.28	**H26, H27 pyridine**	7.90 (s), 9.76 (s)	7.39, 8.27	**H27, H28 pyridine**	7.23 (s), 8.04 (s)	7.36, 8.29
**H42substituted phenyl**	7.69 (s)	7.83	**H40, H41, H42, H43 substituted phenyl**	6.89(d), 7.72(d)	6.99, 8.03	**H41, H42, H43, H44 substituted phenyl**	6.48–7.90 (m)	6.84–8.13 (m)
**H43, H44 substituted phenyl**	6.92(d), 7.35 (d)	6.87, 7.06	**CH29–P proton**	6.09 (s)	6.37	**CH30–P proton**	6.47 (s)	7.30
**CH31–P proton**	6.46 (s)	6.43	**2 × CH2–CH3**	3.97–4.03 (m)	4.22–4.95 (m)	**O–CH3**	3.75 (s)	3.99
**O–CH3**	3.75 (s)	3.72	**2 × CH2–CH3**	1.02 (t)	0.97	**2 × CH2–CH3**	3.83–3.98 (m)	4.04–4.65 (m)
**2 × CH2–CH3**	3.81–4.01 (m)	4.15–4.45				**2 × CH2–CH3**	0.96–1.17 (m)	0.90–1.32 (m)
**2 × CH2–CH3**	1.18 (t)	1.01						

** 3d**			** 3e**			** 3f**		
	Experimental^**a**^	Theoretical^b^		Experimental^**a**^	Theoretical^b^		Experimental^**a**^	Theoretical^b^
**H27, H28 pyridine**	7.89 (s), 8.91 (s)	7.50, 8.28	**H28, H29 pyridine**	7.89 (s), 9.63 (s)	7.37, 8.26	**H24, H25 pyridine**	7.71 (s), 8.08 (s)	7.41, 8.38
**H43, H44** **substituted phenyl**	7.02 (d), 7.39 (d)	7.25, 8.00	**H42, H45, H43, H44 substituted phenyl**	6.72(d), 7.63(d)	6.62, 7.29	**H40 thiophene**	7.89 (s)	8.31
**H45, H46, H47, H48 substituted phenyl**	7.56–8.15 (m)	7.70–7.86 (m)	**CH31–P proton**	6.47 (s)	6.47	**H38, H39 thiophene**	6.95 (d), 7.44 (d)	7.18, 7.58
**CH32–P proton**	5.72 (d)	7.08	**N–(CH3)2**	2.99 (s)	2.82	**CH27–P proton**	5.18 (d)	6.74
**2 × CH2–CH3**	3.84–4.02 (m)	4.03–4.30 (m)	**2 × CH2–CH3**	3.97–4.03 (m)	4.22–4.95 (m)	**2 × CH2–CH3**	3.96–3.99 (m)	4.12–4.31
**2 × CH2–CH3**	1.00 (t), 1.20 (t)	1.10 (t)	**2 × CH2–CH3**	1.02 (t)	0.97	**2 × CH2–CH3**	1.06–1.21 (m)	1.04–1.29 (m)

** 3g**								
	Experimental^a^	Theoretical^b^						
**H26, H27 pyridine**	7.89 (s), 9.44 (s)	7.38, 8.30						
**H40, H41, H42, H43 substituted phenyl**	6.91–8.48 (m)	7.23–8.17 (m)						
**CH29–P proton**	6.44 (s)	6.88						
**2 × CH2–CH3**	3.80–4.02 (m)	4.19–4.56 (m)						
**2 × CH2–CH3**	1.05–1.23 (m)	0.99–1.33						

Every value is expressed in terms of the TMS chemical shift, calculated at the same theoretical level

^a^Experimental values of chemical shifts in this work obtained to 500 MHz in DMSO

^b^Theoretical values of chemical shifts obtained by DFT/B3LYP/6-311G method in DMSO

**Table 7:** Theoretical and experimental ^13^C-NMR chemical shifts (ppm) of **3a**–**g** compounds computed at the level of the DFT/B3LYP/6-311G theory

			**Chemical shifts, δ (ppm)**					
** 3a**			** 3b**			** 3c**		
	Experimental^a^	Theoretical^b^		Experimental^a^	Theoretical^b^		Experimental^a^	Theoretical^b^
**HC–P**	62.24	66.18	**HC–P**	58.38	66.32	**HC–P**	62.01	60.34
**2 × CH2–CH3**	14.23	19.33	**2 × CH2–CH3**	27.72	20.85	**2 × CH2–CH3**	17.11	19.55
**2 × CH2–CH3**	68.74	70.52	**2 × CH2–CH3**	69.91	70.67	**2 × CH2–CH3**	63.89	70.41
**C=N pyridine**	145.27	152.09	**C=N pyridine**	155.04	155.66	**C=N pyridine**	136.66	152.13
**C–NH**	148.78	155.61	**C–NH**	144.72	152.12	**C–NH**	144.67	155.76
**C aromatic**	111.11–129.35	124.17–131.45	**C Aromatic**	114.29–136.31	121.91–138.93	**C Aromatic**	11.12–129.26	117.64–131.70
**C–Cl**	144.93	142.95	**C–Cl**	142.14	142.89	**C–Cl**	135.95	142.93
**OCH3**	56.35	56.61	**C–OH**	163.81	167.16	**OCH3**	56.02	59.34
**C–OCH3**	153.49	156.29				**C–OCH3**	155.06	156.34
**C–OH**	154.95	159.39						

3d			3e			3f		
	Experimental^a^	Theoretical^b^		Experimental^a^	Theoretical^b^		Experimental^a^	Theoretical^b^
**HC–P**	70.03	71.60	**HC–P**	61.88	68.50	**HC–P**	57.45	62.69
**2 × CH2–CH3**	16.20	18.34	**2 × CH2–CH3**	16.72	18.54	**2 × CH2–CH3**	13.56	18.44
**2 × CH2–CH3**	60.22	68.90	**2 × CH2–CH3**	72.06	70.22	**2 × CH2–CH3**	63.81	69.44
**C=N pyridine**	144.17	152.89	**C=N pyridine**	154.43	155.29	**C=N pyridine**	154.89	155.60
**C–NH**	154.54	157.77	**C–NH**	144.62	151.92	**C aromatic**	114.09–136.38	120.30–142.49
**C aromatic**	112.78–136.49	119.33–139.42	**C aromatic**	111.39–132.77	116.71–142.27	**C–Cl**	144.70	142.89
**C–Cl**	139.14	139.82	**C–Cl**	136.47	138.05			
**C–OH**	164.23	163.89	**N–(CH3)2**	44.57	42.34			

3g								
	Experimental^a^	Theoretical^b^						
**HC–P**	66.17	60.69						
**2 × CH2–CH3**	16.92	19.55						
**2 × CH2–CH3**	67.56	70.44						
**C=N pyridine**	154.68	152.13						
**C–NH**	161.91	155.73						
**C aromatic**	127.14–138.87	129.23–142.43						
**C–Cl**	146.04	143.01						
**C–OH**	166.42	162.54						

Every value is expressed in terms of the TMS chemical shift, calculated at the same theoretical level

^a^Experimental values of chemical shifts in this work obtained to 125 MHz in DMSO

^b^Theoretical values of chemical shifts obtained by DFT/B3LYP/6-311G method in DMSO

## Conclusion

Diethyl(((3,5-dichloropyridin-2-yl)amino)(Aryl)methyl)phosphonate, **3a**–**g** were synthesized and their structures were elucidated using different spectroscopic methods.The phosphonate compound which has naphthyl substituent, **3d**, clarified the most potentantimicrobial and antioxidant activity among all other phosphonates. Moreover,Molecular docking revealed that compound **3d**, could be a targeted anticancer agent because it has a good docking score and its nitrogen, oxygen atoms, and phenyl moieties form hydrogen bonds, and hydrophobic and electrostatic interactions with crucial residues within the binding pocket of Drp-1 target protein. Confirming the docking inhibitory results, the in-vitro anti-cancer activities, against panel of cancer cell lines were interpreted the suppressive impact of compound **3d**. Also,the theoretical FT-IR data had been calculated to discover the characteristic vibration frequencies of the compounds which show a good correlation with experimental data. Moreover, the theoretical nuclear magnetic resonance (NMR) of the synthesized phosphonates was synthesized and a good correlation was obtained between theoretical and experimental data. Thus, our study recommended the usage of compound **3d** as newly antimicrobialagent and a targeted inhibitory candidate against dynamin-related protein 1 (Drp-1) mitochondrial fission protein that responsible for cancer therapy. Also, we recommended further synthesis of novel α-am containg more than one phenyl and hydroxyl groups that may investigate a powerful antimicrobial and antitumor impact.

## Supplementary Information

Supplementary Material 1.

## Data Availability

• The microbial strains were provided from Microbial Type Culture Collection (MTCC) and all deposited codes were added two gram-positive bacteria (*Bacillus subtilis* (*#**MTCC* NO 441) and *Staphylococcus aureus* (#*MTCC* NO 96). as well as two Gram-negative bacteria (*Pseudomonas aeruginosa (#**MTCC* NO 1688*)*, *Escherichia coli*(#*MTCC* NO 452)). Two fungi (*Candida albicans*(#*MTCC* NO 183) and *Aspergillus flavus*(#*MTCC* NO 1344)). • The cell lines were provided from the American Type Culture Collection (ATCC) via VACSERA, Cairo, Egypt, and all accession codes were added Mammary gland (MCF-7;# *ATCC* HTB-22), colorectal adenocarcinoma (Caco-2; *# **ATCC* HTB-37), hepatocellular carcinoma (HepG-2; #*ATCC* HB-8065), and human lung fibroblast (WI-38; # *ATCC* CCL-75). • The datasets generated and/or analyzed during the current study are available in: Macromolecule protein structure, can be deposited in the worldwide protein data bank repository, (https://www.rcsb.org/structure/1ZWS). • the geometry of investigated compounds. The density functional theory (DFT) was utilized to simulate chemical processes and predict material characteristics using the hybrid density functional technique B3LYP combination with a 6-311G++(d, p) basis set for examined substances. The data supporting the current study's findings are available from the corresponding author upon reasonable request.
