# Supplementary material for: Inhibition of DRP-1 mitochondrial mitophagy and fission by novel α-aminophosphonates bearing pyridine: synthesis, biological evaluations, and computer-aided design
Source: BMC Chem. 2024 Sep 18;18(1):174. doi: 10.1186/s13065-024-01268-2 (PMC11409709; doi:10.1186/s13065-024-01268-2)
Supplement: Supplementary file 1 — Supplementary Material 1. [file 13065_2024_1268_MOESM1_ESM.docx]

**Inhibition of DRP-1 mitochondrial mitophagy and fission by novel α-Aminophosphonates bearing pyridine: Synthesis, Biological evaluations, and Computer-aided Design**

**Hend A. Hekal^1*^, Maha M. Salem^2^, Hayam A. Abd El Salam^3^**

^1^Chemistry Department, Faculty of Science, Tanta University, 31527Tanta, Egypt

^2^Biochemistry Division, Chemistry Department, Faculty of Science, Tanta University, Tanta, Egypt

*^3^*Green Chemistry Department, National Research Centre, Dokki, Giza 12622, Egypt.

**Emails:** [**hend.hekal@science.tanta.edu.eg**](mailto:hend.hekal@science.tanta.edu.eg)**,** [**maha_salem@science.tanta.edu.eg**](mailto:maha_salem@science.tanta.edu.eg)**,** [**yooma_nrc82@yahoo.com**](mailto:yooma_nrc82@yahoo.com)

**Running title:** Biological assessments of α-aminophosphonates bearing pyridine nucleus.

***Corresponding and Proof:**

Dr. Hend A. Hekal

Chemistry Department

Faculty of Science

Tanta University

Tanta, Egypt

Email: [**hend.hekal@science.tanta.edu.eg**](mailto:hend.hekal@science.tanta.edu.eg)

**Section S1: Materials and instrumentation**

Melting points were measured on an Electrothermal melting point apparatus. IR spectra were recorded on a PerkinElmer 317 grating IR spectrophotometer using KBr pellets. NMR spectra were measured with a JEOL E.C.A- 500 MHz (^13^C: *125 MHz, ^1^H: *500 MHz) spectrometer. ^1^H and ^13^C NMR spectra were measured using SiMe_4_ as an internal reference. Mass spectrometry was performed on a JEOL JMSAX 500 spectrometer. The appropriate precautions in handling moisture-sensitive compounds were considered. Solvents were dried by standard techniques. Thin-layer chromatography (TLC) used Merck 0.2 mm silica gel 60 F_254_ anal aluminum plates. Elemental analyses were conducted at the Microanalysis Laboratory, Cairo University, Cairo, Egypt; their values agreed favorably with the calculated ones. The biological evaluation of the products was carried out at the Pharmacognosy Department, Mansoura University,
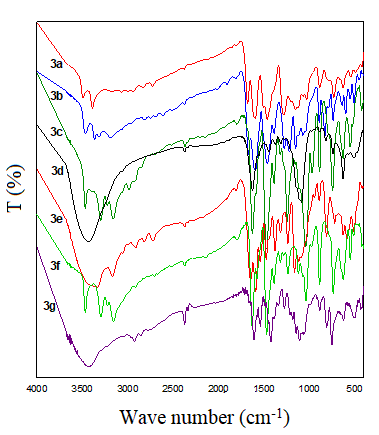
Egypt.

**Fig.S1.** FT-IR spectra of α-aminophosphonate compounds **3a-3g**.


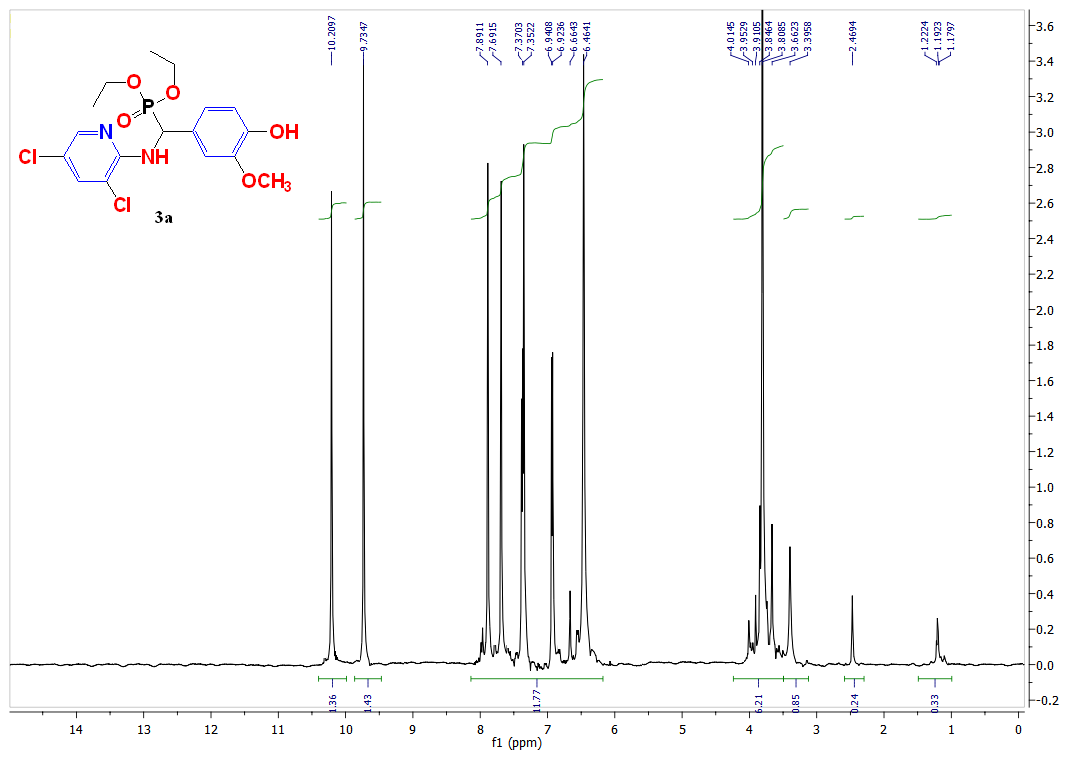


**Fig.S2.** ^1^H-NMR of α-aminophosphonate compound **3a**.

**Fig.S3.**
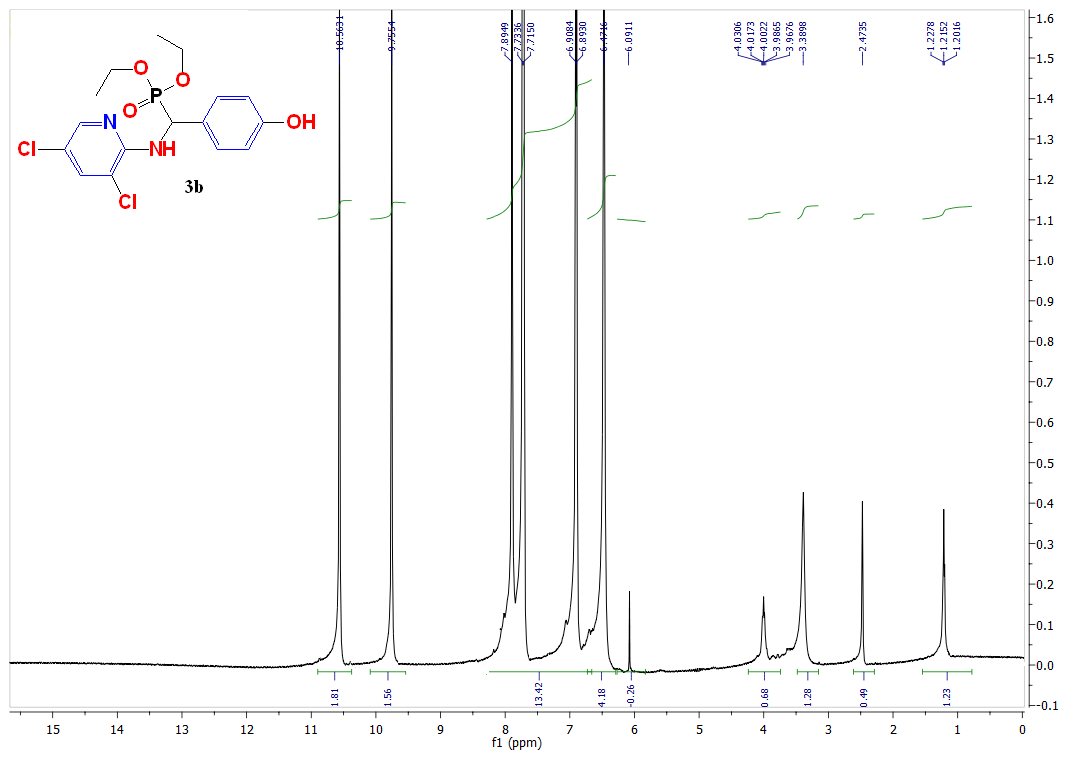
^1^H-NMR of α-aminophosphonate compound **3b**.

**Fig.S4.**
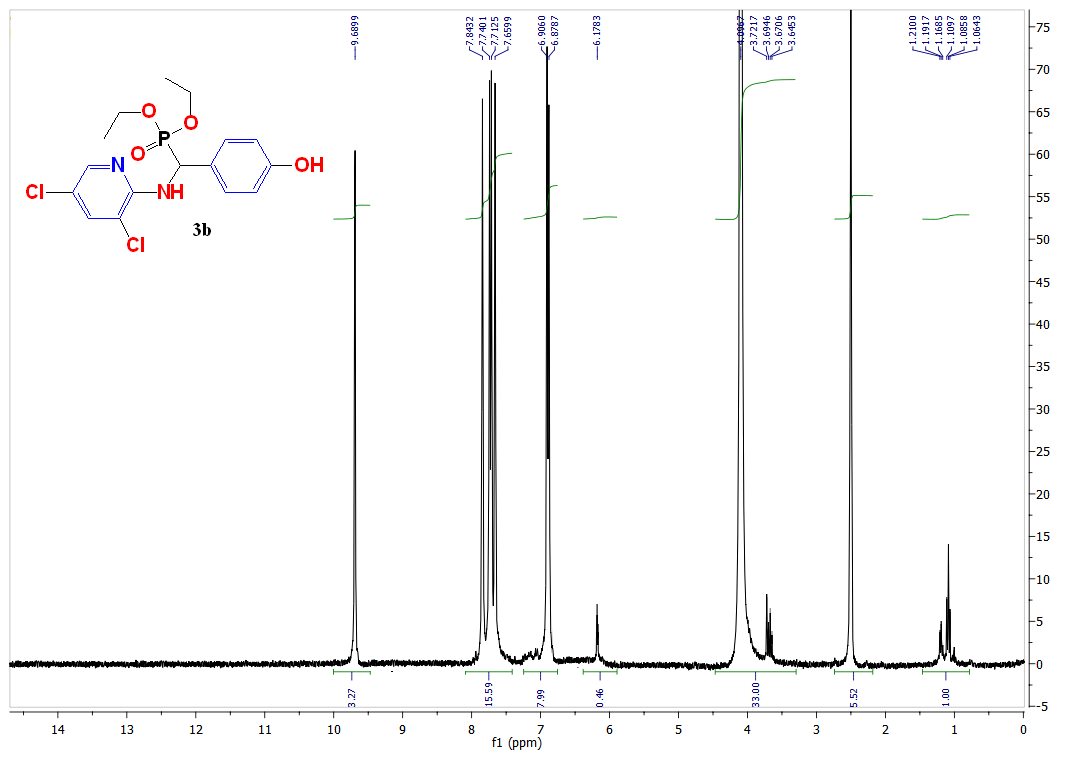
D_2_O-^1^H-NMR of α-aminophosphonate compound **3b**.

**Fig.S5.** ^1^H-NMR of α-aminophosphonate compound **3c**.


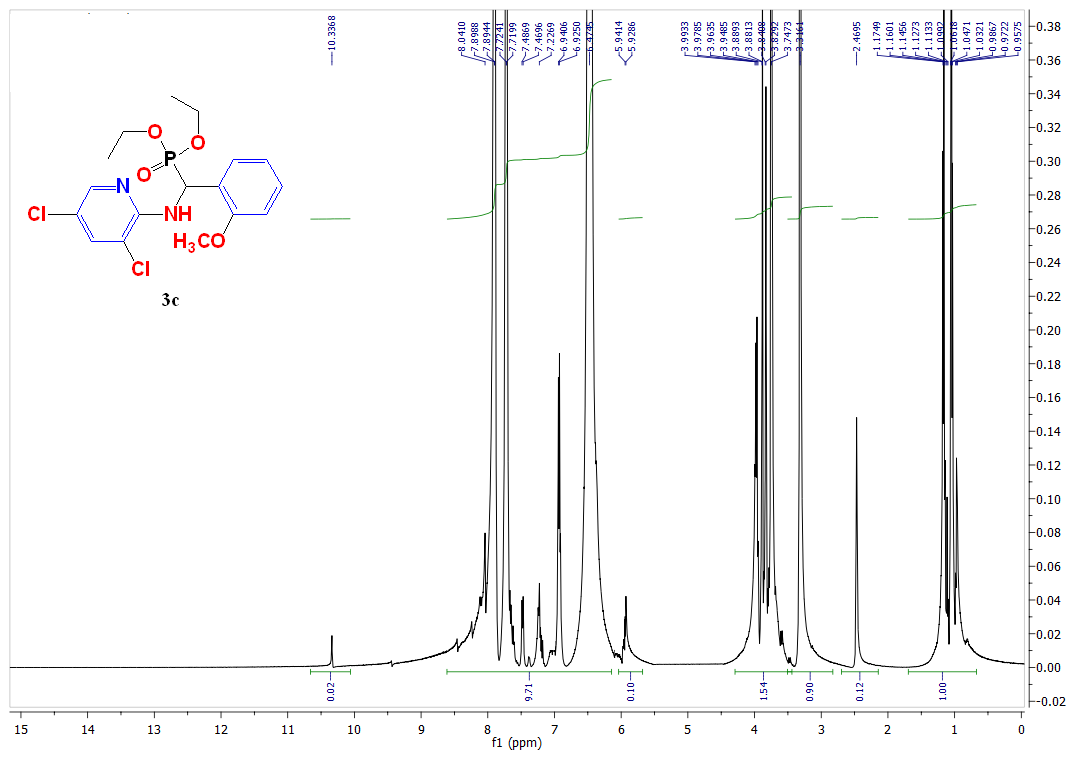


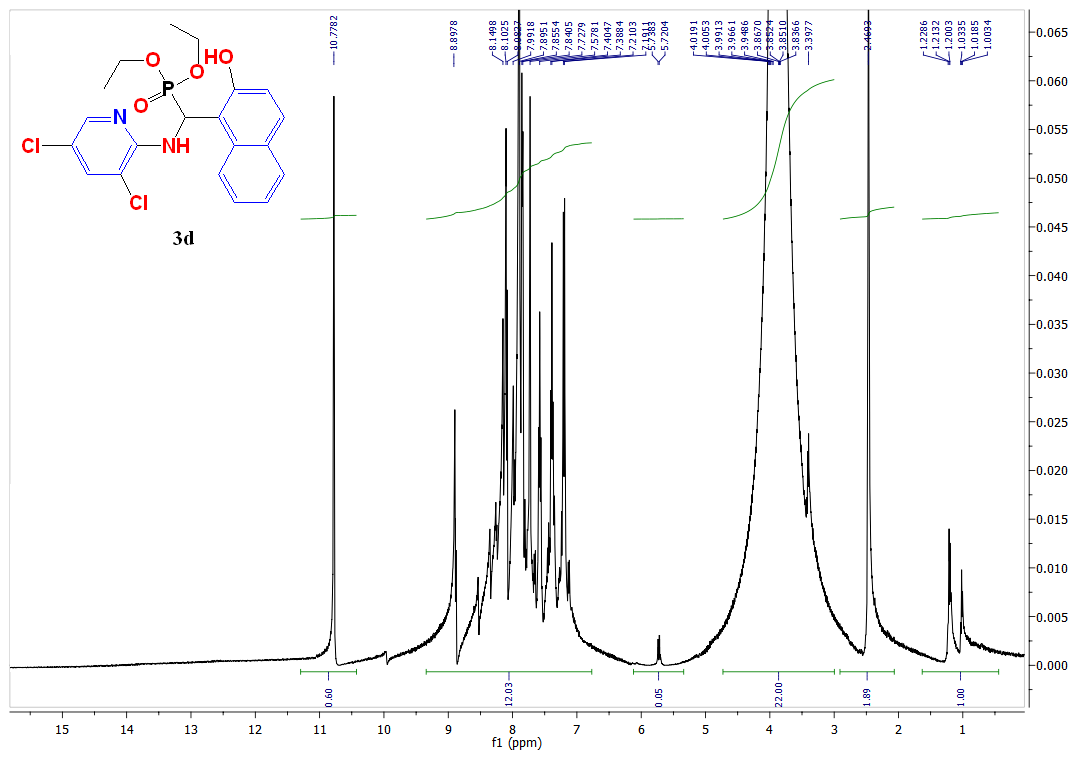


**Fig.S6.** ^1^H-NMR of α-aminophosphonate compound **3d**.


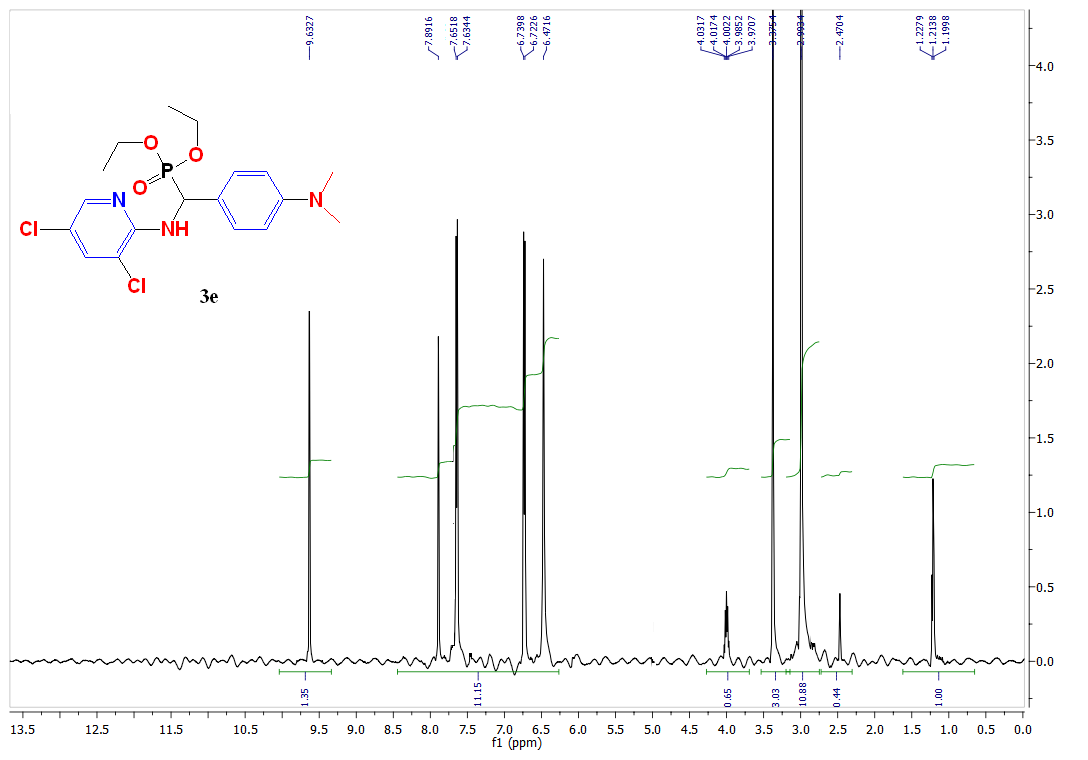


**Fig.S7.** ^1^H-NMR of α-aminophosphonate compound **3e**.

**Fig.S8.** ^1^
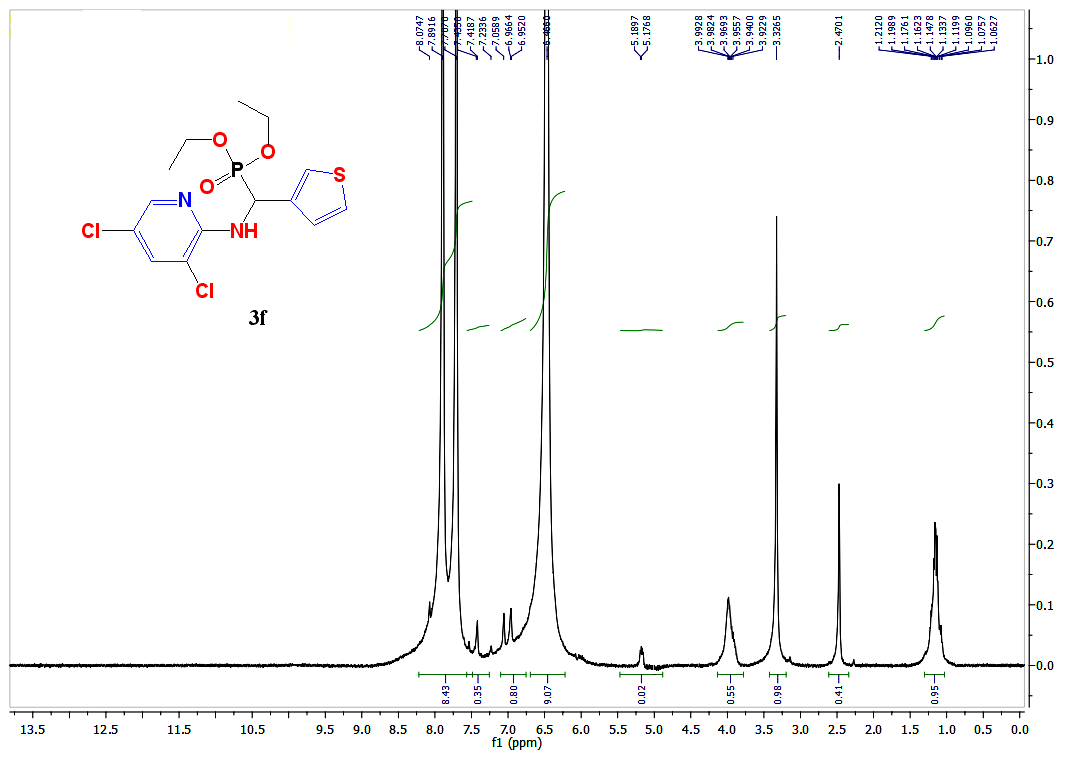
H-NMR of α-aminophosphonate compound **3f**.


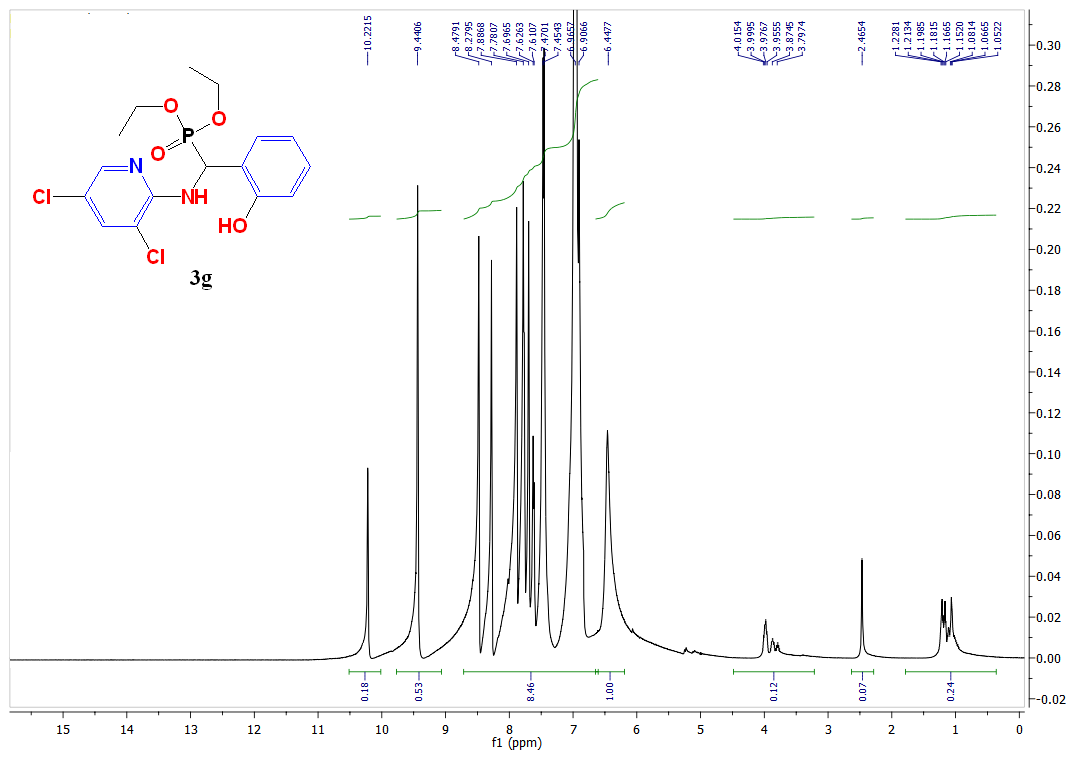


**Fig.S9.** ^1^H-NMR of α-aminophosphonate compound **3g**.


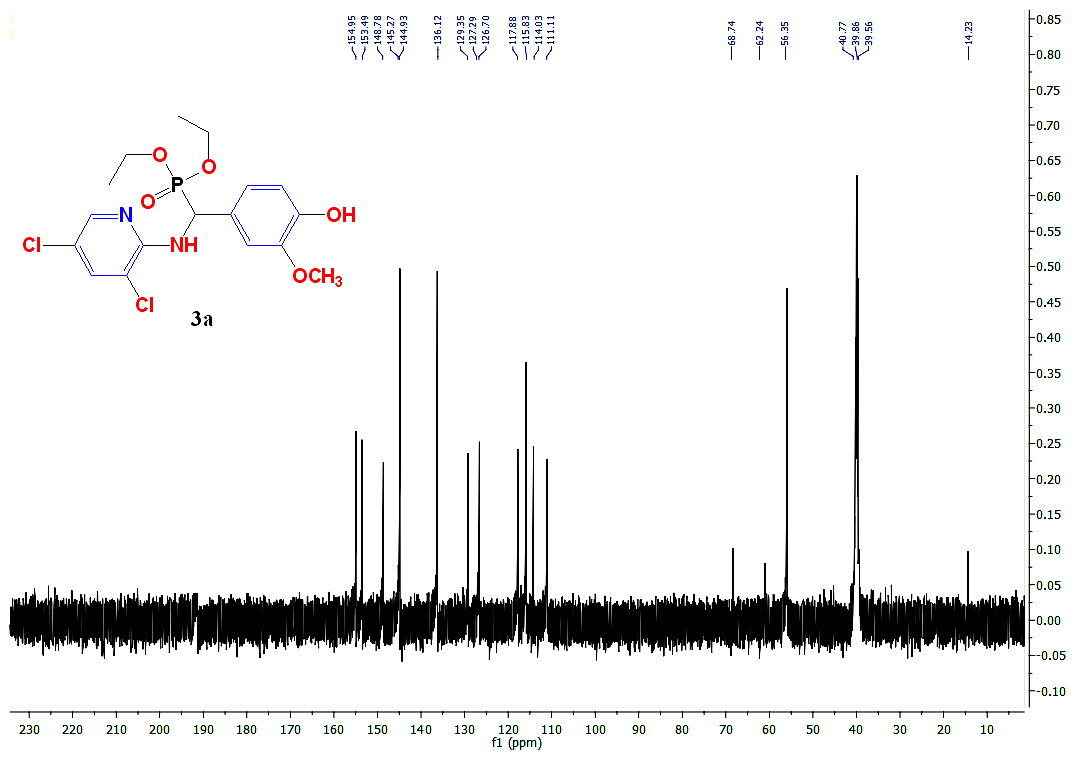


**Fig.S10.** ^13^C-NMR of α-aminophosphonate compound **3a**.


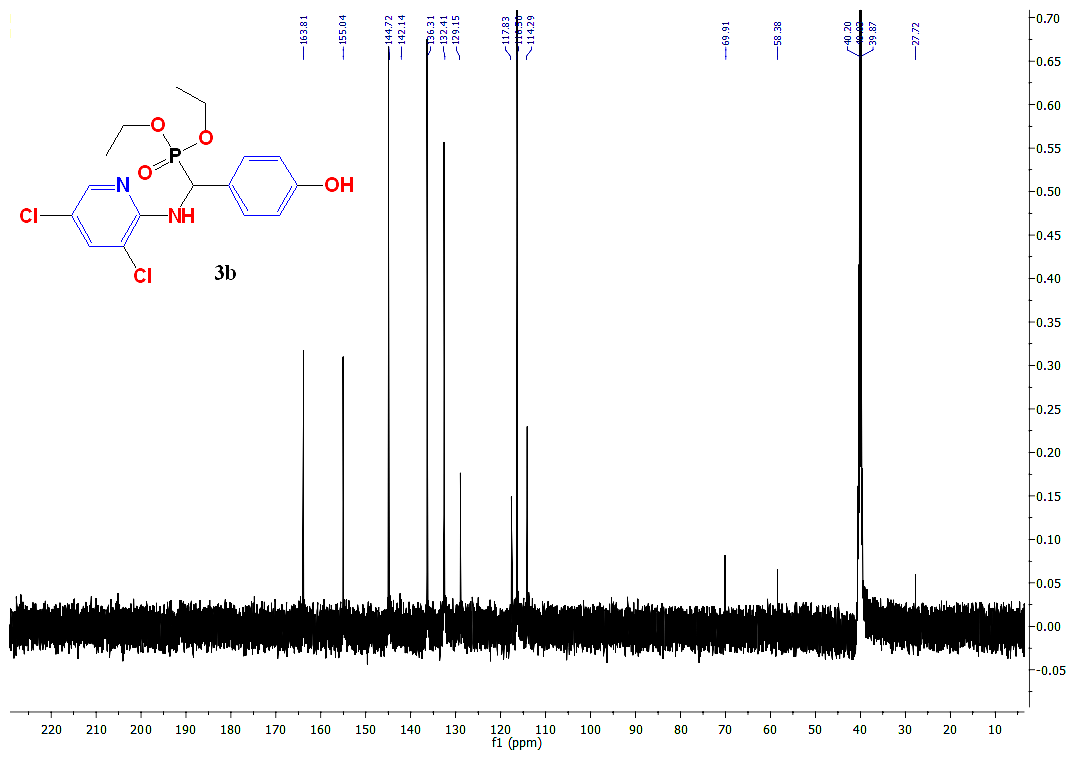


**Fig.S11.** ^13^C-NMR of α-aminophosphonate compound **3b**.


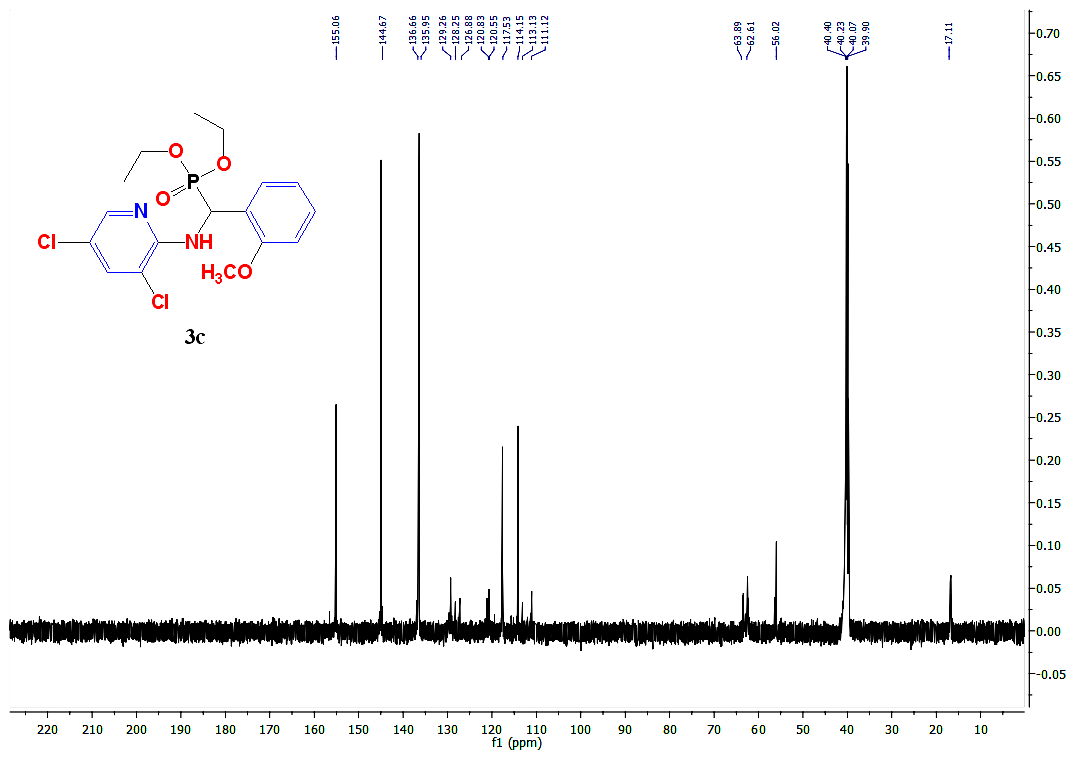


**Fig.S12.** ^13^C-NMR of α-aminophosphonate compound **3c**.


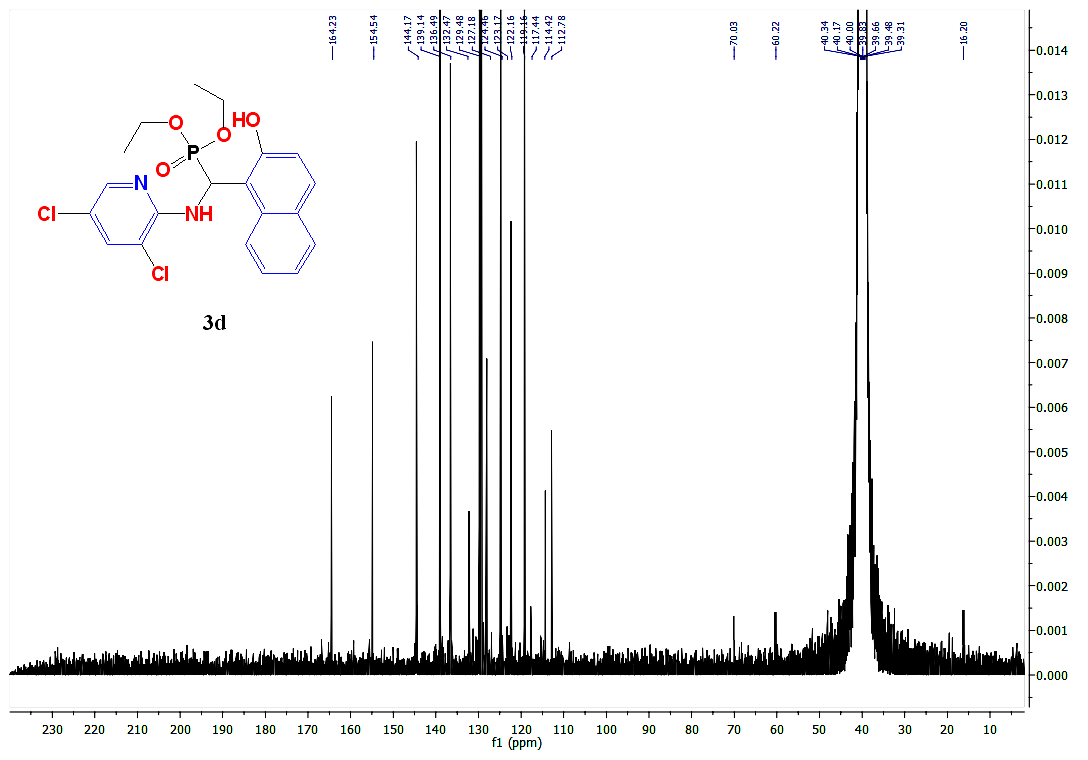


**Fig.S13.** ^13^C-NMR of α-aminophosphonate compound **3d**.


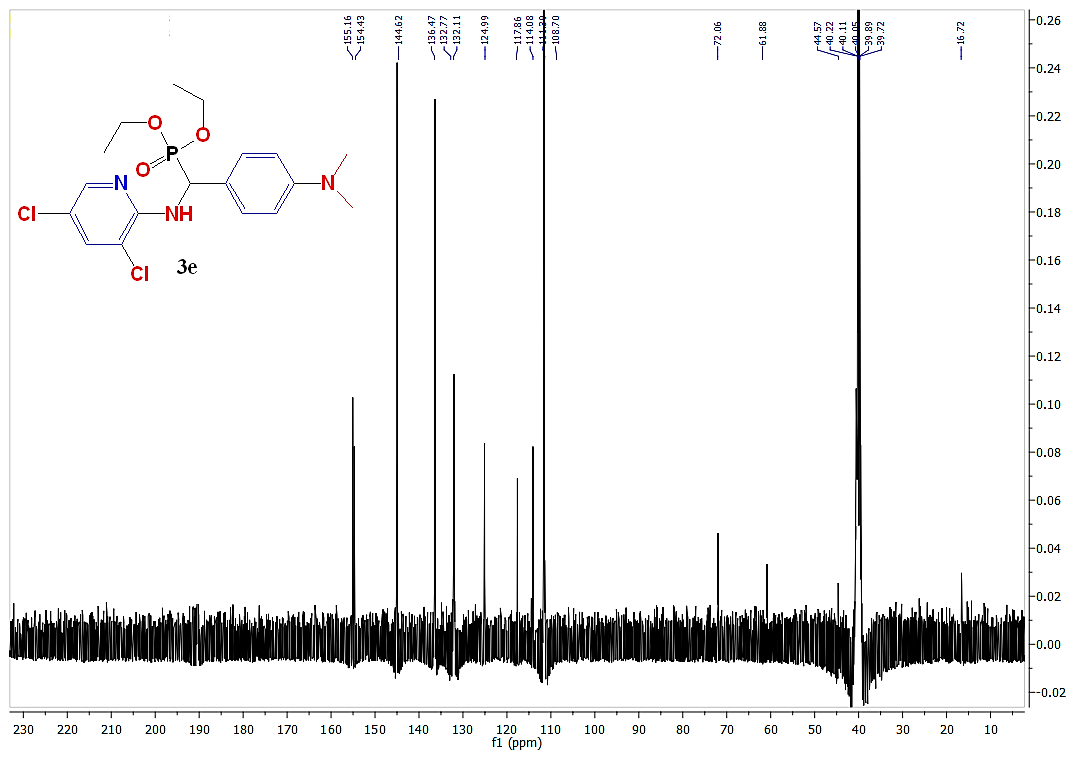


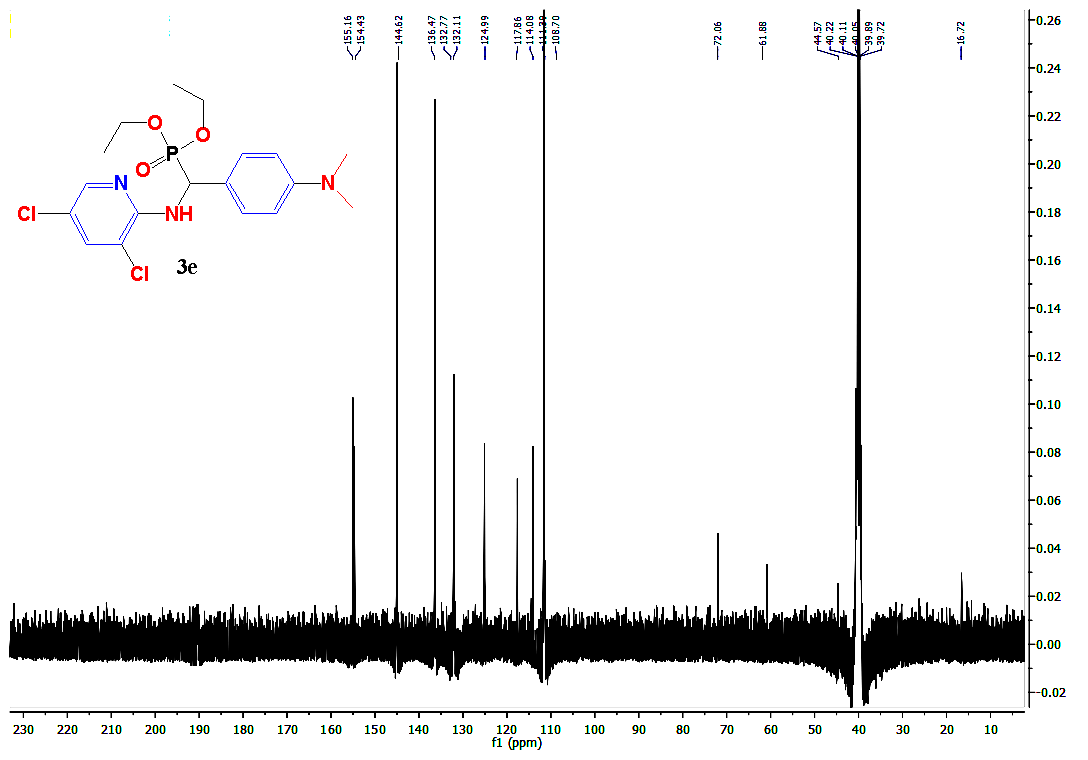


**Fig.S14.** ^13^C-NMR of α-aminophosphonate compound **3e**.


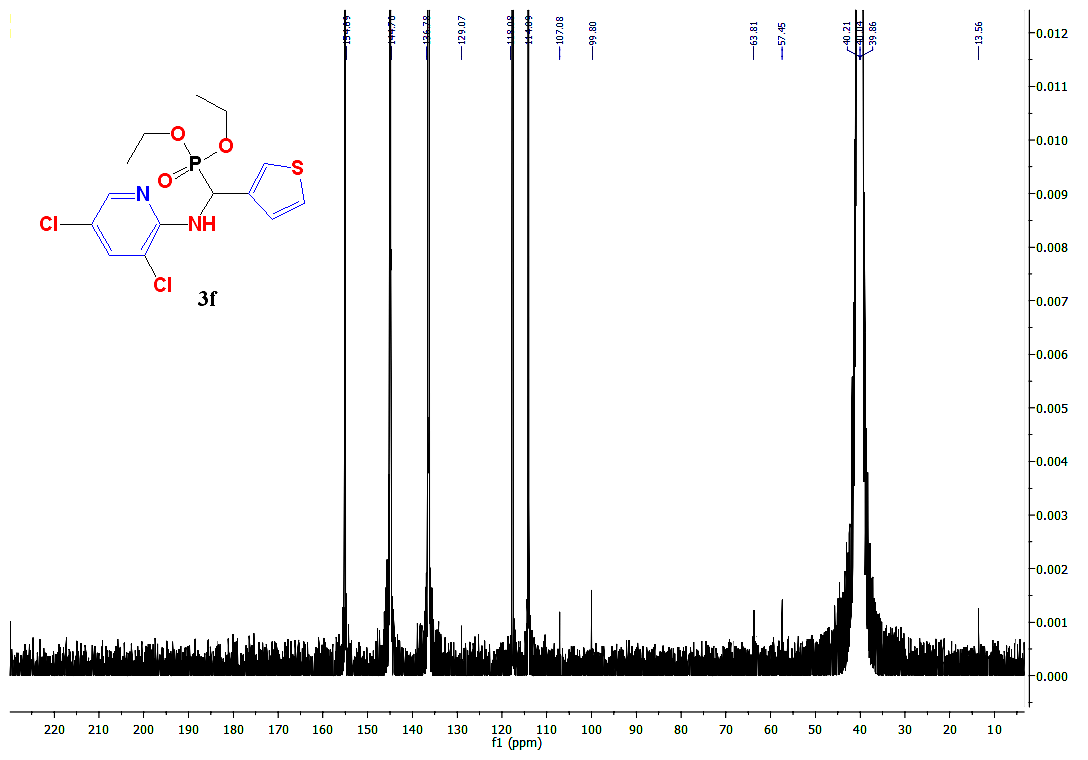


**Fig.S15.** ^13^C-NMR of α-aminophosphonate compound **3f**.


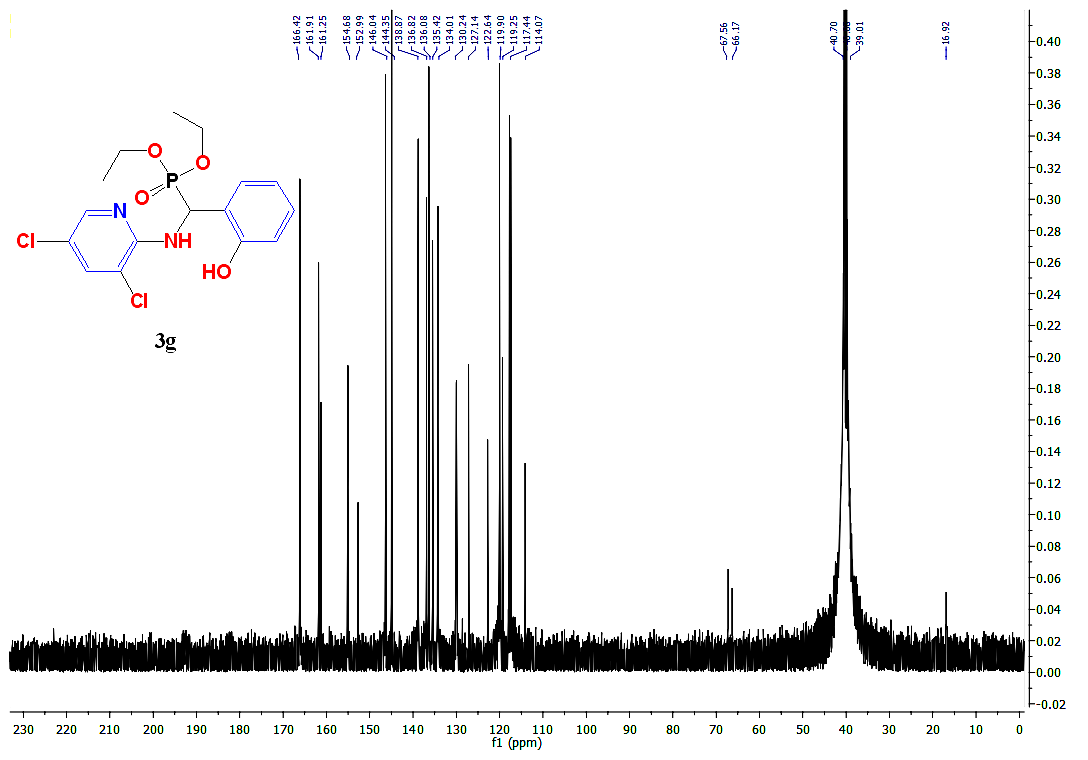


**Fig.S16.** ^13^C-NMR of α-aminophosphonate compound **3g**.


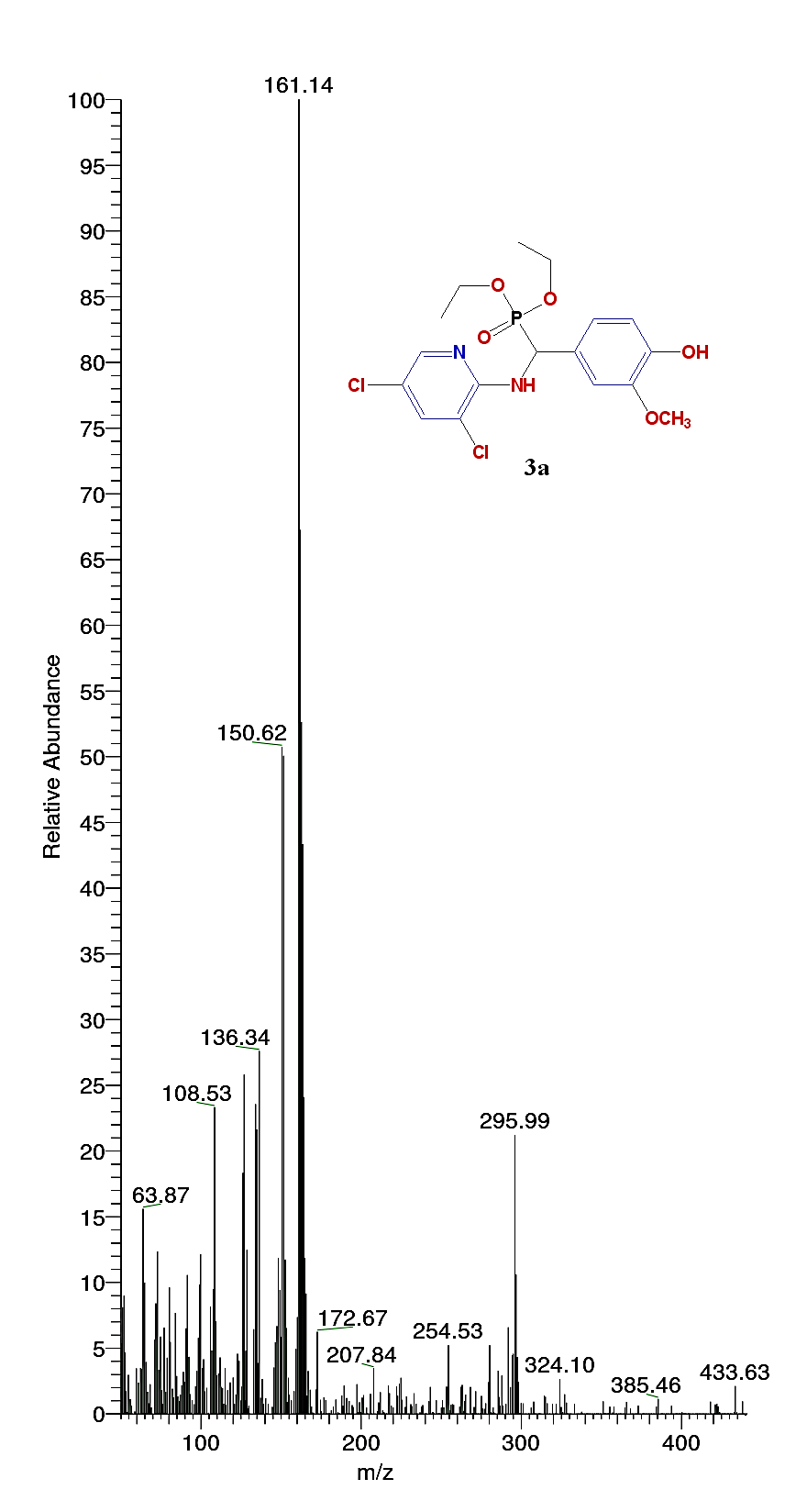


**Fig.S17.** Mass of α-aminophosphonate compound **3a**.


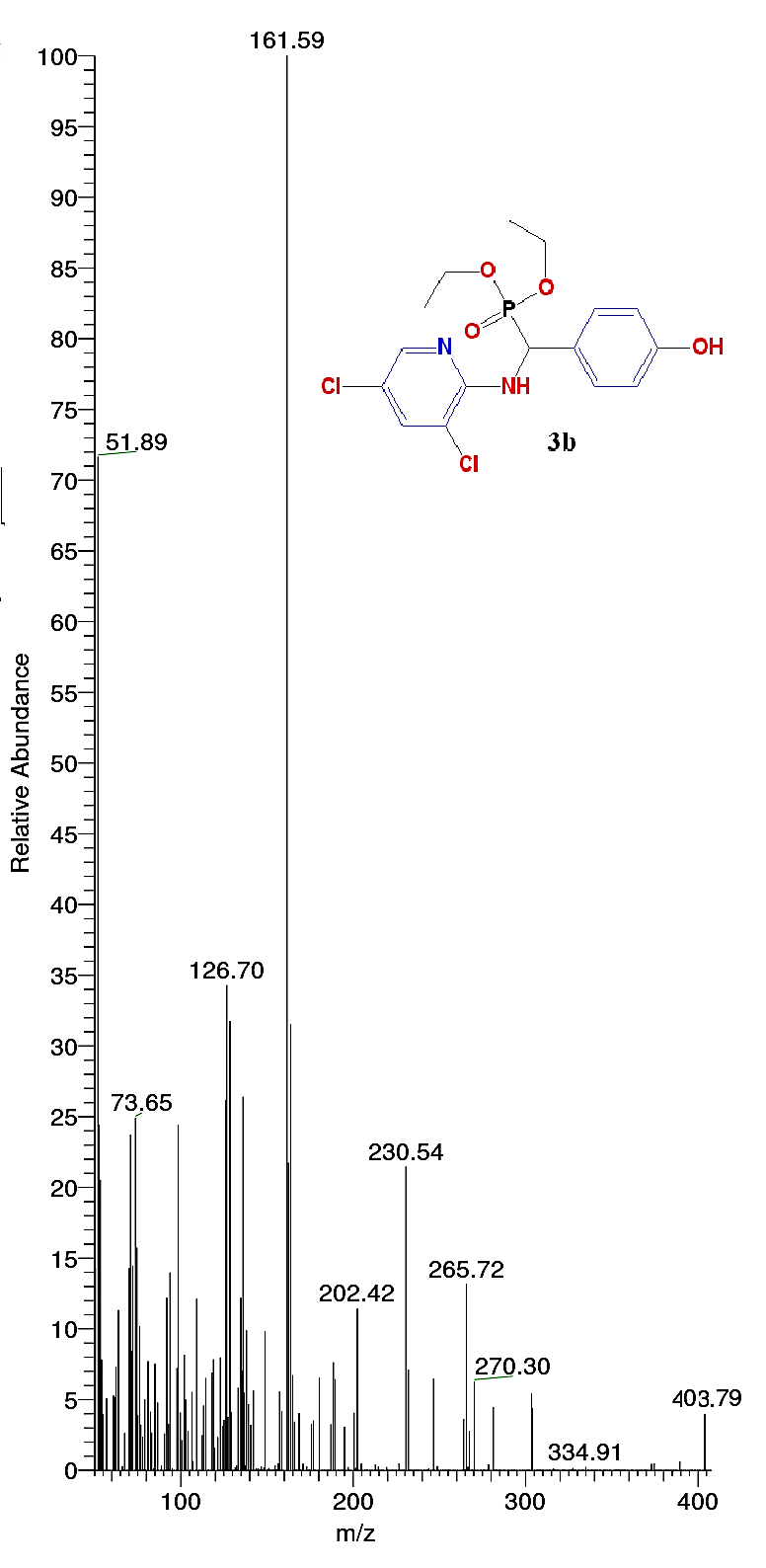


**Fig.S18.** Mass of α-aminophosphonate compound **3b**.


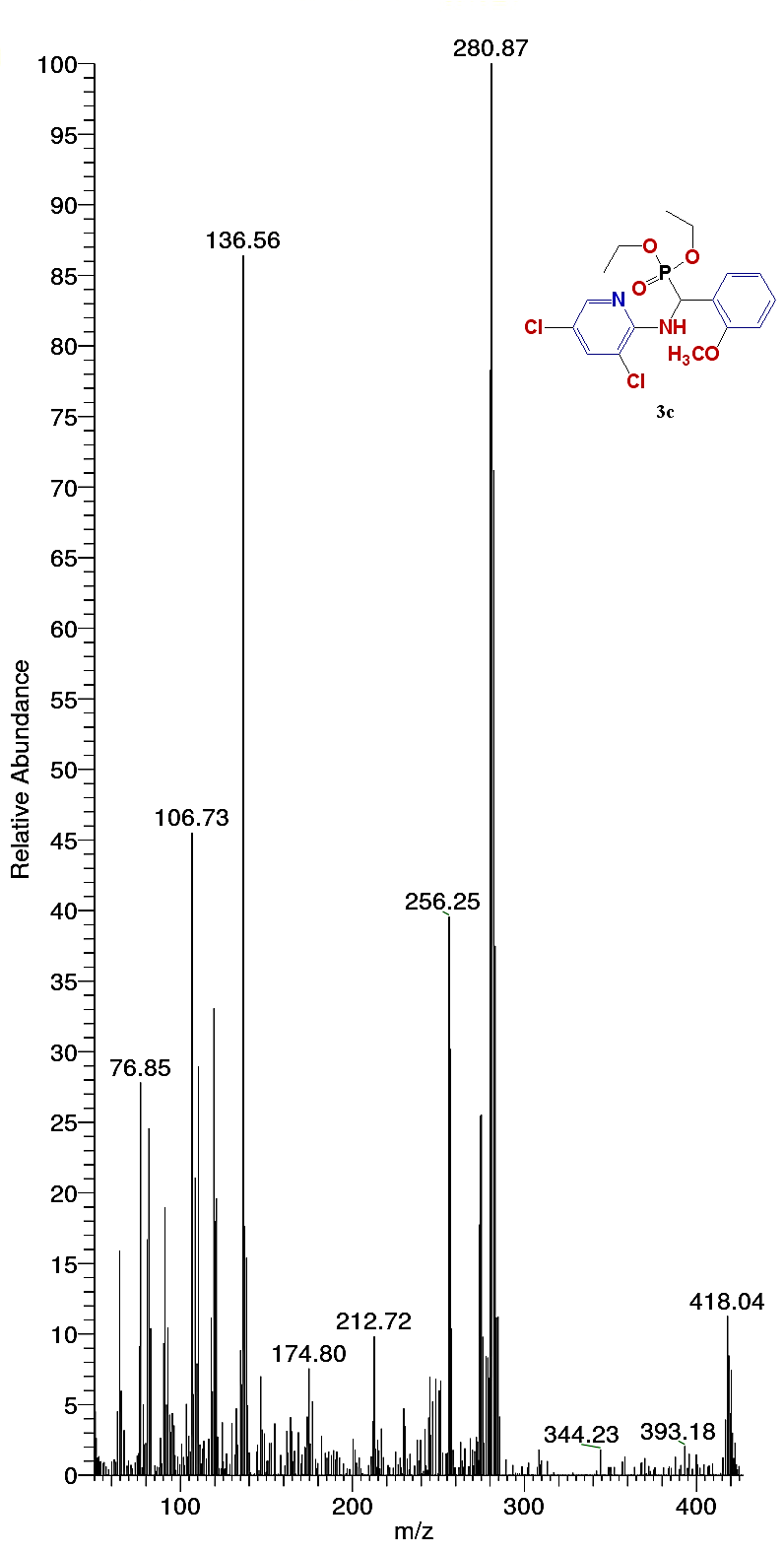


**Fig.S19.** Mass of α-aminophosphonate compound **3c**.


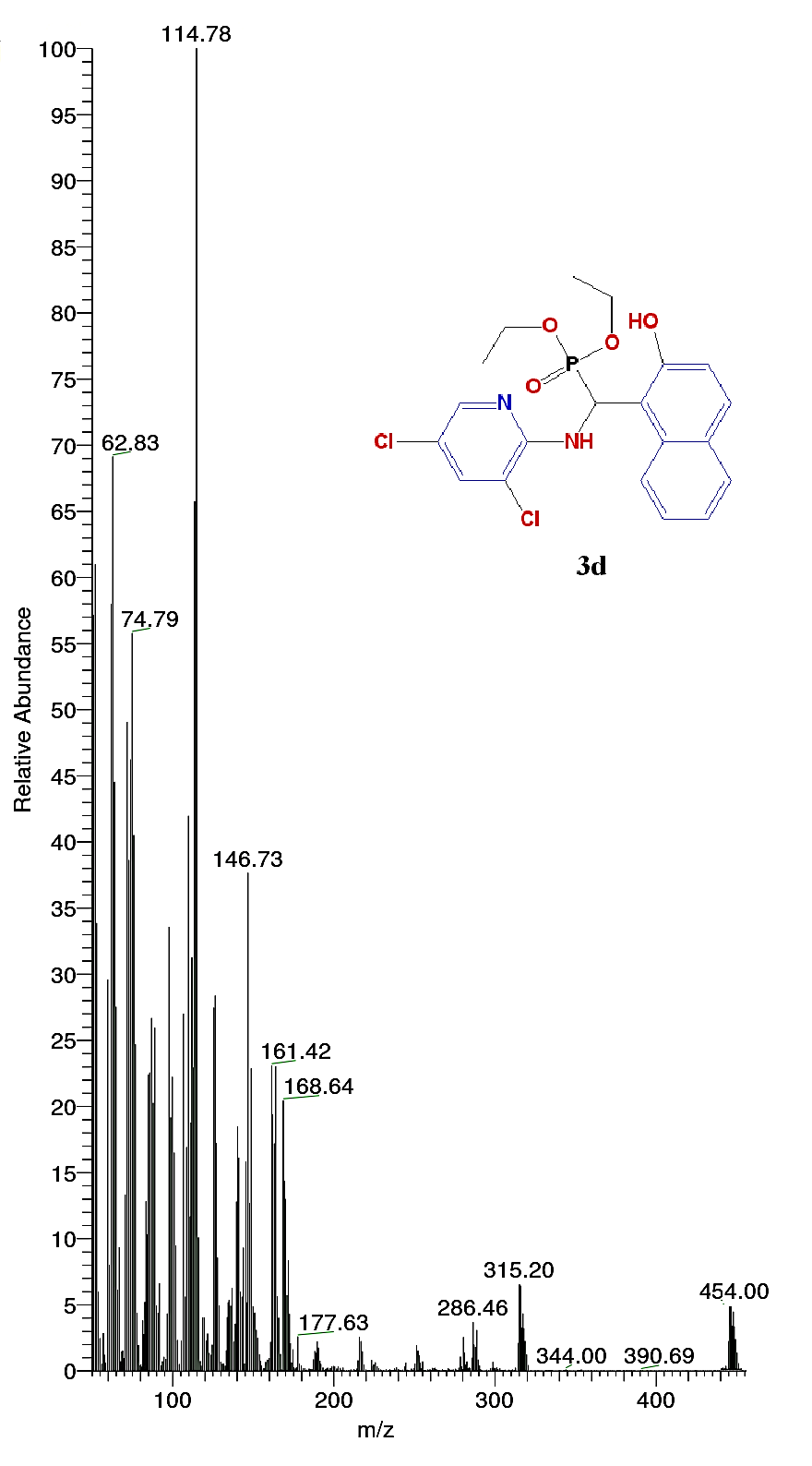


**Fig.S20.** Mass of α-aminophosphonate compound **3d**.


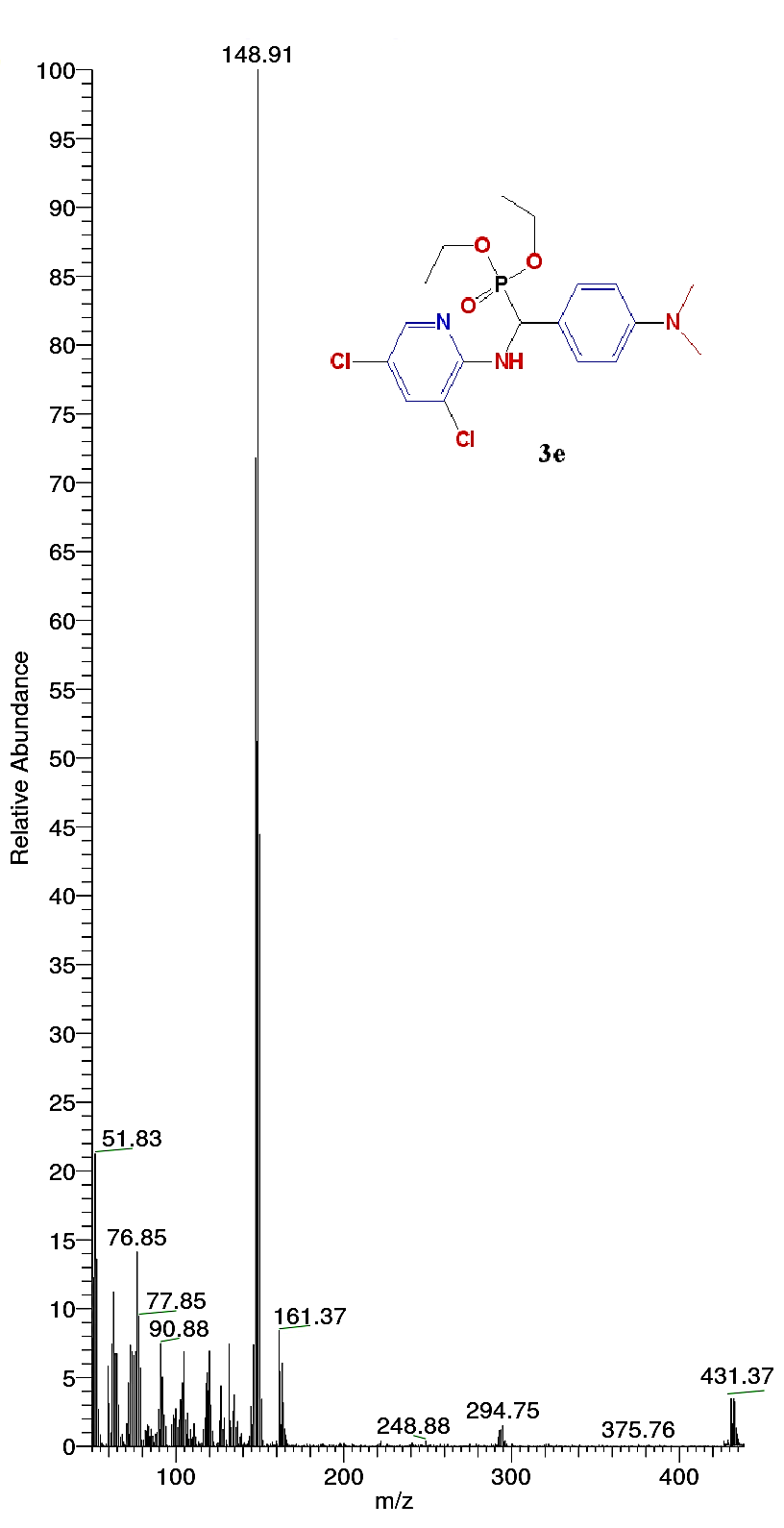


**Fig.S21.** Mass of α-aminophosphonate compound **3e**.


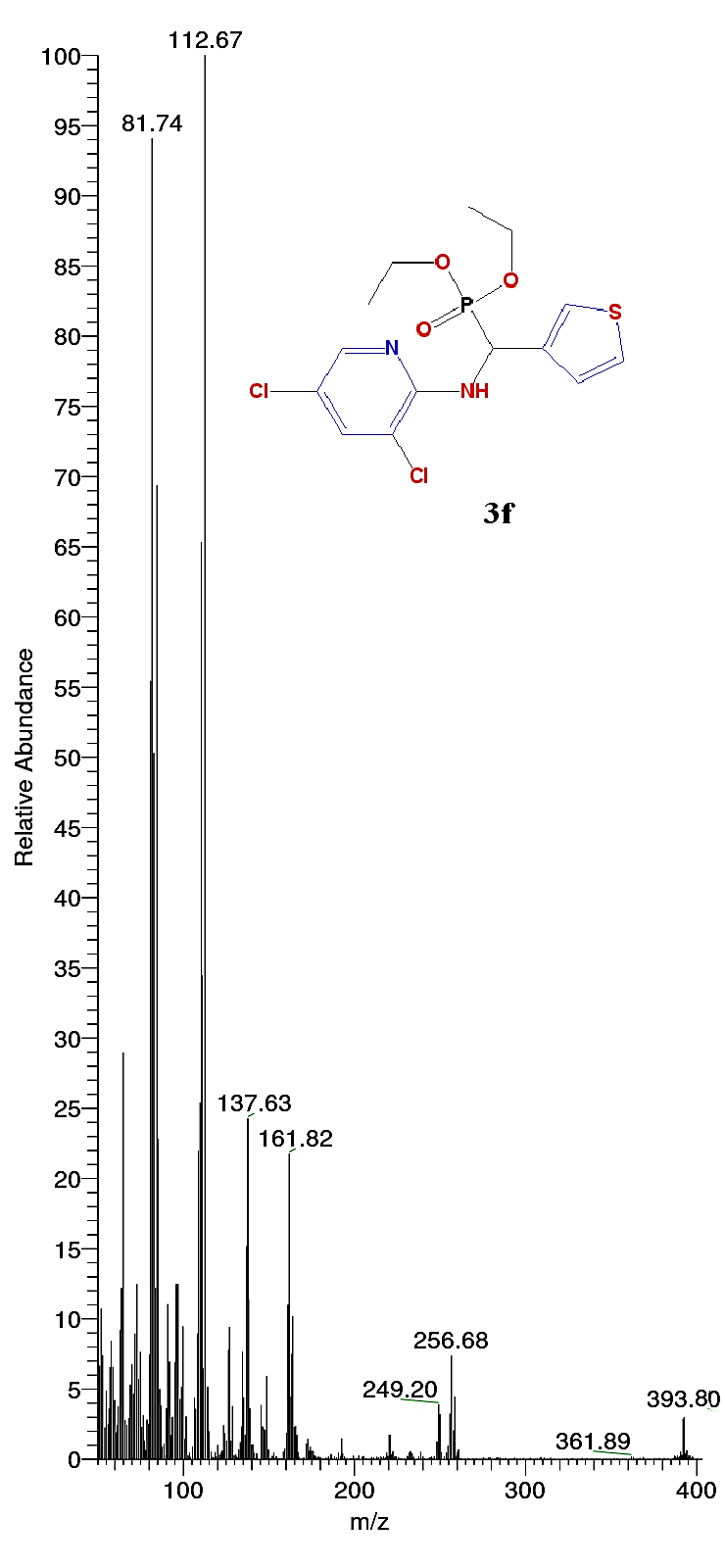


**Fig.S22.** Mass of α-aminophosphonate compound **3f**.


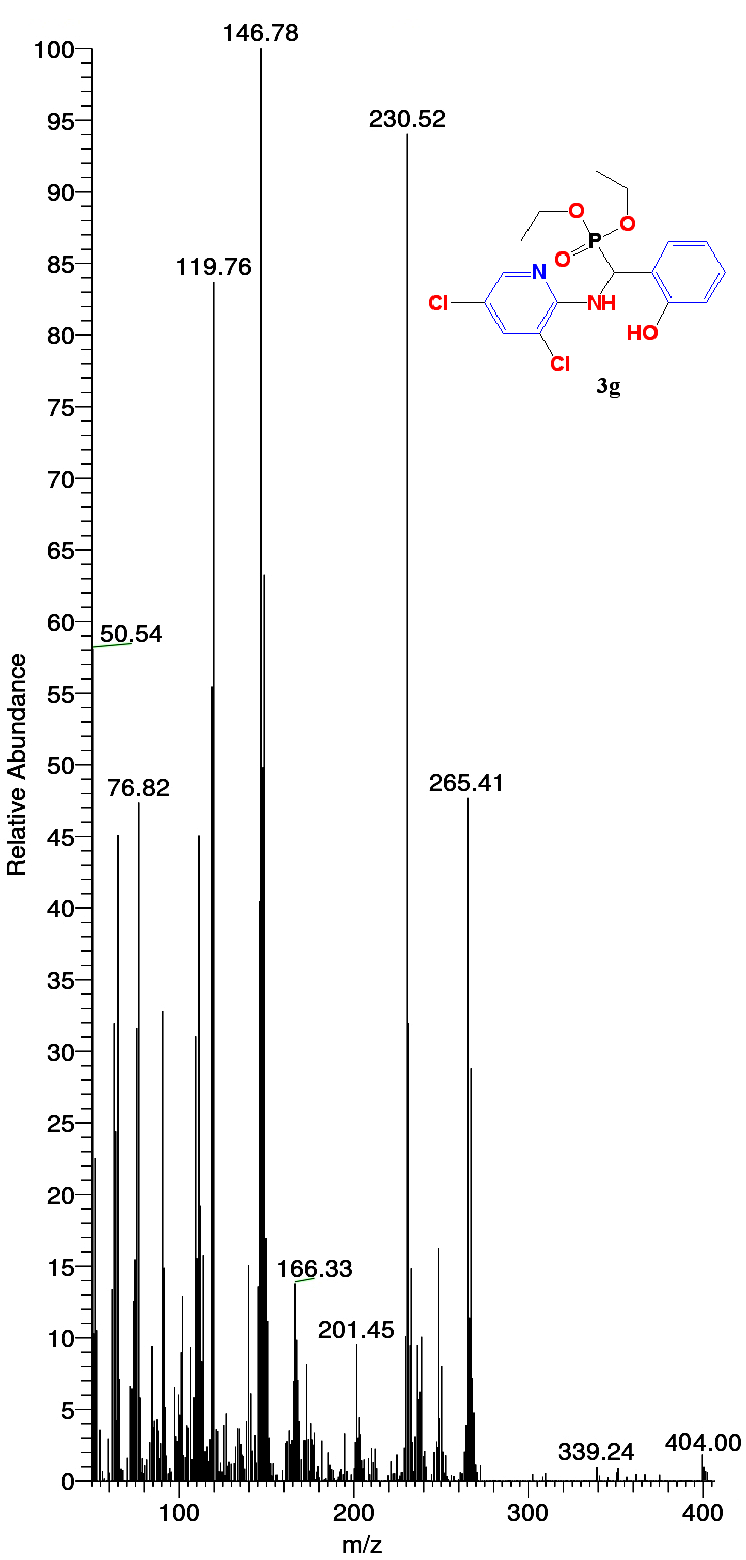


**Fig.S23.** Mass of α-aminophosphonate compound **3g**.
